# Uncertainty and Predictiveness Modulate Attention in Human Predictive Learning

**DOI:** 10.1037/xge0000991

**Published:** 2020-11-30

**Authors:** Chang-Mao Chao, Anthony McGregor, David J. Sanderson

**Affiliations:** 1 Department of Psychology, Durham University

**Keywords:** learning, attention, associability, prediction error, conditioning

## Abstract

Attention determines which cues receive processing and are learned about. Learning, however, leads to attentional biases. In the study of animal learning, in some circumstances, cues that have been previously predictive of their consequences are subsequently learned about more than are nonpredictive cues, suggesting that they receive more attention. In other circumstances, cues that have previously led to uncertain consequences are learned about more than are predictive cues. In human learning, there is a clear role for predictiveness, but a role for uncertainty has been less clear. Here, in a human learning task, we show that cues that led to uncertain outcomes were subsequently learned about more than were cues that were previously predictive of their outcomes. This effect occurred when there were few uncertain cues. When the number of uncertain cues was increased, attention switched to predictive cues. This pattern of results was found for cues (1) that were uncertain because they led to 2 different outcomes equally often in a nonpredictable manner and (2) that were used in a nonlinear discrimination and were not predictive individually but were predictive in combination with other cues. This suggests that both the opposing predictiveness and uncertainty effects were determined by the relationship between individual cues and outcomes rather than the predictive strength of combined cues. These results demonstrate that learning affects attention; however, the precise nature of the effect on attention depends on the level of task complexity, which reflects a potential switch between exploration and exploitation of cues.

There is a reciprocal relationship between attention and learning. Attention determines which cues are selected for processing and are learned about. Learning, however, subsequently influences attention. Therefore, it is important that the theoretical analyses of both attention and learning account for the interplay between the two processes. Research on associative learning in animals has found that, depending on the procedure, learning can have different, opposing effects on attention. One effect is the *predictiveness effect* in which cues that are better predictors of outcomes than other cues (e.g., a noise signaling food in a Pavlovian conditioning procedure is a better predictor than the incidental contextual cues) gain attention (Mackintosh, 1975). Another effect is the *uncertainty effect* in which cues that are uncertain by virtue of either being novel or leading to a number of outcomes (e.g., food or no food) in a variable manner receive a high level of attention compared with cues for which the outcomes are certain (e.g., a cue that always leads to food; Pearce & Hall, 1980). The effects are opposite effects. A predictive cue is a cue that correlates with a particular outcome and, therefore, there is some certainty associated with consequences of the cue. An uncertain cue is a cue that is nonpredictive or less predictive than other cues either due to being novel and its consequence unknown or because its presentation does not correlate reliably with an outcome. The outcome may be present on some occasions but not others or the cue may lead to different outcomes or different quantities of an outcome in a random manner.

Both the predictiveness and uncertainty effects may reflect potential heuristics for efficient information processing given that attention is a limited resource. The predictiveness effect results in increased attention to stimuli that are relevant for a particular task and decreased attention to stimuli that are irrelevant or redundant. The uncertainty effect results in attention being reduced for cues that have already been learned such that attentional resources can be maximized for cues whose consequences are unknown. The difference between the two effects has been suggested to potentially reflect the difference between exploration and exploitation of cues for achieving optimality (Beesley, Nguyen, Pearson, & Le Pelley, 2015). The trade-off between devoting attention to predictive cues and using attention to reduce uncertainty occurs in any form of categorization task. For example, an experienced radiologist will be efficient at diagnosis by paying attention to features of images that are good predictors of pathology and ignoring features that are not relevant for diagnosis (utilizing predictiveness). However, attention must also be paid to features of images that have uncertain diagnostic properties to avoid misdiagnosis (utilizing uncertainty).

The fact that learning influences attention means that information processing is biased by previous experience. Although this may lead to more efficient information processing, it may also lead to biases in learning and behavior that do not necessarily reflect the true statistical properties of experienced events. For example, the formation of stereotypes of social groups can be the result of differences in the previous degree of predictiveness of social group characteristics for evaluatively neutral information (Le Pelley et al., 2010; Spiers, Love, Le Pelley, Gibb, & Murphy, 2017). This suggests that the attentional biases that occur because of learning are likely to have a profound effect on many aspects of cognition such as beliefs, attitudes and decision making. Furthermore, an impaired ability to acquire attentional biases because of learning may lead to abnormal cognitive processes. Attentional deficits in neuropsychiatric disorders such as schizophrenia have been proposed to reflect a failure to reduce attention to irrelevant, nonpredictive cues (Haselgrove et al., 2016; Le Pelley, Schmidt-Hansen, Harris, Lunter, & Morris, 2010). Failure to reduce attention to irrelevant cues correlates with the positive symptoms of schizophrenia suggesting that delusions and hallucinations may be a consequence of abnormal information processing (Kapur, 2003; Morris, Griffiths, Le Pelley, & Weickert, 2013).

In the field of associative learning in animals, two theories have been developed to account for predictiveness and uncertainty: the predictiveness effect is accounted for by Mackintosh (1975) and the uncertainty effect is accounted for by Pearce and Hall (1980). Although these theories make some opposing predictions, they describe different mechanisms for the effect of learning on attention. Therefore, it is possible that they account for separate, dissociable processes. Indeed, hybrid models that combine the predictiveness and uncertainty mechanisms have been developed in order to capture this notion (Le Pelley, 2004; Pearce & Mackintosh, 2010).

## Theories of Attention in Associative Learning

Both the theories of Mackintosh (1975) and Pearce and Hall (1980) proposed that the salience of a cue is changed as a consequence of prediction error. Prediction error occurs when the outcome of a cue is not expected. The strength of an association between a cue and an outcome represents the extent to which a cue predicts the outcome. As associative strength increases prediction error decreases and learning ceases when the outcome is fully predicted. Although prediction error can be large for unexpected outcomes (e.g., at the start of training of a cue–outcome association), it can also be large when a fully expected outcome is omitted. The Mackintosh model proposes that on a given trial, the salience of the cue that is the best predictor of the outcome increases and the salience of other cues that are present decrease. Therefore, prediction error is calculated for each cue, independent of the associative strength of the other cues present, and the cue with the smallest prediction error gains attention. Changes in associative strength for individual cues on a trial are calculated by the following equation:

$$\Delta {\text{V}}_{\text{A}}=\alpha_{\text{A}}\cdot \text{θ}\cdot (\lambda –{\text{V}}_{\text{A}})\text{.}$$ 1

Prediction error is represented by the discrepancy between the current associative strength of Cue A (V_A_) and the maximum associative strength that can be supported by the outcome (λ). The salience of the outcome (θ) and the salience of Cue A (α_A_) determine the rate at which the current prediction error drives changes in learning on a given trial. Changes in α_A_ on a given trial are governed by the following rule:

$$\begin{array}{l} \Delta \alpha_{\text{A}}>0\text{ if }\left|\lambda –{\text{V}}_{\text{A}}\right|<\left|\lambda –{\text{V}}_{\text{X}}\right|\\ \Delta \alpha_{\text{A}}<0\text{ if }\left|\lambda –{\text{V}}_{\text{A}}\right|\geq \left|\lambda –{\text{V}}_{\text{X}}\right| \end{array}\text{.}$$ 2

**Figure 1 fig1:**
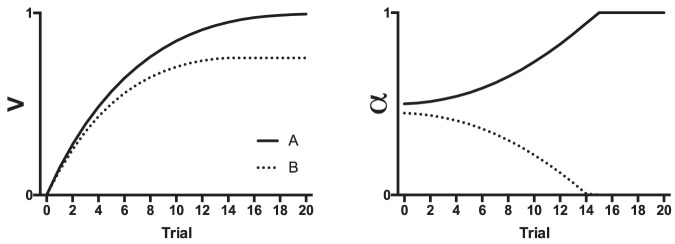
Simulation of the Mackintosh (1975) model for two cues (A and B) that have a different initial values of α before conditioning in compound (AB→outcome). For the simulation the starting value of α for Cue A was 0.5 and 0.45 for Cue B. λ equalled 1, and θ, the learning rate parameter determined by the unconditioned stimulus was 0.3. Changes in associative strength (V) over training are shown in the left panel and changes in α (the associability of the cue) are shown in the right panel. Over training, Cue A gains more associative strength than does Cue B. Whereas α increases for Cue A, it decreases for Cue B over training.

The prediction error for Cue A (λ − V_A_) is compared with the prediction error for all the other cues present on a trial (λ − V_X_). If the error is smaller for Cue A than all the other cues, then α_A_ increases but if it is the same or greater then it decreases. The consequences of these rules for changes in associability are clear when considering a situation in which two cues with different initial values of alpha are conditioned in compound (e.g., AB; see Figure 1). If we assume that alpha is higher for Cue A than for Cue B, then—because the initial increase in associative strength on Trial 1 will be higher for Cue A than for Cue B—Cue A will be a better predictor of the outcome than Cue B. This results in alpha increasing for Cue A and decreasing for Cue B. The difference in associative strength between the cues drives the difference in alpha further over training and the difference in alpha determines the subsequent maximum associative strength that the cues eventually achieve.[Fig-anchor fig1]

The Pearce–Hall model, in contrast to the Mackintosh model, does not assume that changes in attention to a cue are governed by the individual prediction error for that cue, but the summed error for all cues present on a trial. The following equation determines changes in associative strength on a given trial:

$$\Delta {\text{V}}_{\text{A}}=\alpha_{\text{A}}\cdot \text{S}\cdot \lambda \text{.}$$ 3

S and lambda are determined by the intensity of the cue and the outcome, respectively. Here, alpha reflects specifically the associability of the cue, that is, how readily the cue is able to form associations with other stimuli. Alpha changes with experience determined by the extent of prediction error according to the following equation:

$$\alpha_{\text{n}}=\lambda –\Sigma {\text{V}}_{\text{n}–\text{1}}\text{.}$$ 4

**Figure 2 fig2:**
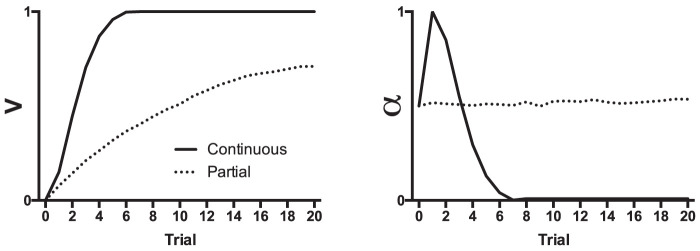
Simulation of Pearce and Hall (1980) model for continuous reinforcement, in which a cue is reinforced on every trial, and partial reinforcement, in which a cue is reinforced on 50% of trials. The starting value of alpha, the associability of the cue, was 0.5. Lambda equaled 1, and S, the learning rate parameter determined by the unconditioned stimulus (US) was 0.3. The learning rate for the absence of the unconditioned stimulus was also set at 0.3. Changes in associative strength (V) over training are shown in the left panel and changes in α are shown in the right panel. Continuous reinforcement results in greater acquisition of associative strength (V) compared with partial reinforcement. Although there is an initial increase in alpha with continuous reinforcement, alpha rapidly decreases as associative strength increases. With partial reinforcement, however, alpha remains high. Simulations were run using the CAL-R Pearce–Hall Simulator (Grikietis, Mondragón, & Alonso, 2016).

Prediction error is calculated as the discrepancy between lambda (the maximum associative strength supported by the unconditioned stimulus) and the combined associative strength of all the stimuli present on a trial (ΣV). Therefore, in contrast to the Mackintosh model that calculates prediction error for each cue (individual error term), the Pearce–Hall model assumes that prediction error is determined by the additive strength of all cues (summed error term). The associability of a cue on a given trial (*n*) is determined by the size of prediction error on the preceding trial (*n* − 1). As prediction error decreases (i.e., as associative strength increases), alpha decreases. Importantly, alpha is determined by the size of prediction error regardless of whether it is positive (due to the surprising presence of an outcome) or negative (due to the surprising absence of an outcome). Therefore, partial reinforcement, in which a cue is sometimes paired with an outcome and sometimes not, leads to alpha remaining high over training. This scenario is modeled in Figure 2. Cues A and B were paired with reinforcement on separate trials, but Cue A was reinforced on every trial, and Cue B was reinforced on 50% of trials. Although the associative strength of Cue A is higher than that of Cue B, the alpha for Cue A reduces in comparison to Cue B over training in line with decreases in the size of prediction error. Alpha for Cue B remains high over training.[Fig-anchor fig2]

Although both models assume that the initial level of alpha is determined by the salience of a cue and the amount of attention that it receives, the Mackintosh model and Pearce–Hall model propose that changes in alpha specifically determine how readily a cue will be learned (i.e., a cue’s associability). Therefore, the experiments that have been used as support for either the Mackintosh (1975) or Pearce and Hall (1980) models have assessed changes in attention by measuring how rapidly new learning is acquired with the cues (e.g., Hall & Pearce, 1979; Mackintosh & Little, 1969). This is done by pairing the previously experienced cues with new outcomes and measuring the extent of learning of the new cue–outcome associations. Although the two theories make opposing predictions, due to the wealth of evidence for both accounts, the results may be reconciled by assuming that individual prediction error and summed prediction error separately lead to predictiveness and uncertainty effects and under particular conditions one effect may outweigh the other (Haselgrove, Esber, Pearce, & Jones, 2010; Le Pelley, 2004; Pearce & Mackintosh, 2010).

By describing potential mechanisms for the interaction between learning and attention, the Mackintosh (1975) and Pearce and Hall (1980) theories have been able to provide accounts for a wide range of phenomena. The original impetus for the theories was to account for selective learning effects, often referred to as cue competition effects, such as blocking (Arcediano, Matute, & Miller, 1997; Kamin, 1969) and overshadowing (Pavlov, 1927; Prados, 2011) in which animals and humans learn about some cues but not others. In addition they provide accounts of the effect of latent inhibition in which preexposure to a cue retards acquisition of learning (Lubow & Moore, 1959), a procedure widely used as a test of selective attention in the assessment of the cognitive impairments in psychopathology (Lubow, 1997). The Mackintosh (1975) and Pearce and Hall (1980) theories provide alternative accounts of these phenomena, but whereas the two theories make similar predictions for some effects, such as cue competition (e.g., they both predict that a blocked cue suffers from a reduction in attention), they each have individual successes in other situations reaffirming the idea that they describe distinct processes. For example, the predictiveness effect has been used to explain the paradoxical, inverse base rate effect in categorization learning (Kruschke, 2001) in which a compound of stimuli for which the individual stimuli have previously been associated with different categories is attributed as belonging to a rare, less frequently experienced category rather than a more common category (Medin & Edelson, 1988). Recently, the role of uncertainty, as described by the Pearce and Hall (1980) model has been applied to the analysis of the circumstances that produce habitual and goal-directed responding (Bouton, Broomer, Rey, & Thrailkill, 2020; Thrailkill, Trask, Vidal, Alcala, & Bouton, 2018). There is also evidence that the processes underlying predictiveness and uncertainty effects reflect dissociable neural substrates (Nasser, Calu, Schoenbaum, & Sharpe, 2017). Neural signals in the basolateral amygdala mimic the course of prediction errors in learning as determined by the Pearce and Hall (1980) model (Esber et al., 2012; Roesch, Calu, Esber, & Schoenbaum, 2010; Roesch, Esber, Li, Daw, & Schoenbaum, 2012). Increases in associability that occur as consequence of uncertainty require the amygdala (Holland & Gallagher, 1993), whereas decrements in associability depend on the hippocampus (Han, Gallagher, & Holland, 1995). In contrast, changes in associability that depend on predictiveness require the prelimbic region of the medial prefrontal cortex (Sharpe & Killcross, 2014, 2015).

## The Role of Predictiveness and Uncertainty in Human Associative Learning

In human associative learning, a role for predictiveness in determining attention has also been found (e.g., Le Pelley & McLaren, 2003), but a role for uncertainty has received less support. Similar to the procedures in the animal literature, changes in attention as a consequence of learning have been assessed by measuring the extent of new learning with cues. In a recent review Le Pelley, Mitchell, Beesley, George, and Wills (2016) cite 18 articles demonstrating that such procedures reveal an effect of predictiveness on attention. In contrast, a role for uncertainty, as measured by associability (how rapidly a cue is learned about), is less clear. Griffiths, Johnson, and Mitchell (2011) showed that prior learning of a predictive relationship between a cue and a moderate outcome reduced the ability of the cue to become predictive of a larger outcome compared with a cue whose consequences were uncertain due to receiving a number of extinction trials in which the moderate outcome was not presented. A similar study, however, failed to find support for a role of uncertainty in attention (Packer, Siddle, & Tipp, 1989). Furthermore, Le Pelley et al. (2016) stated that unpublished attempts to replicate the findings of Griffiths et al. (2011) have not been successful. Recently, however, Easdale, Le Pelley, and Beesley (2017) have shown that sudden switches in the level of uncertainty may increase associability.

Rather than measuring changes in associability, some studies have examined the effect of uncertainty on overt attention, as measured by eye gaze. Hogarth, Dickinson, Austin, Brown, and Duka (2008) found that participants showed greater fixation of eye gaze for a cue that was uncertain cue due to leading to an outcome (an auditory stimulus) on only 50% trials compared with a cue that was predictive due to leading to the outcome on 100% of trials. This effect, however, is not always replicated (Austin & Duka, 2010). Beesley et al. (2015) found that participants spend a greater proportion of time fixating on uncertain cues within a trial than on predictive cues. There was not, however, any advantage of uncertain cues over predictive cues in a subsequent test of associability. Indeed, in the test of associability, the opposite was found, with previously predictive cues learned about more than previously uncertain cues. This has led to the suggestion that uncertainty may affect levels of attention to all cues generally, rather than leading to stimulus-specific changes in attention and associability (Beesley et al., 2015; Le Pelley et al., 2016).

The lack of behavioral evidence for a role of uncertainty in attention in human associative learning is at odds with research demonstrating neural correlates of uncertainty in the human brain. For example, Li, Schiller, Schoenbaum, Phelps, and Daw (2011) found that patterns of activity to cues were sensitive to the absolute prediction error associated with the cue (i.e., the discrepancy between the outcome and the anticipation of the outcome, independent of whether the discrepancy was positive or negative). This neural correlate of unsigned prediction error mimics the calculation of error for determining uncertainty as described by Pearce and Hall (1980). It is possible that the behavioral procedures used to date have not been sensitive enough to detect an effect of uncertainty. Alternatively, the lack of behavioral evidence for uncertainty may suggest that although uncertainty is encoded at some level, it does not impact on attention. If this is the case it would suggest a divergence between humans and nonhuman animals in the learning mechanisms that affect attention.

In the present study, we report a series of experiments that demonstrate that nonpredictive, uncertain cues do receive more attention than predictive cues, under particular conditions, as measured by the extent to which the cues can enter into associations with new outcomes, in a human learning procedure. We examined changes in associability (the learning rate parameter for a cue) rather than more explicit measures of attention such as eye gaze due to the assumptions of the Mackintosh (1975) and Pearce and Hall (1980) models that alpha determines learning rate. Our starting point for this line of work was an experiment (Experiment 1) that was similar in design to an experiment reported by Livesey, Thorwart, De Fina, and Harris (2011), in which cues that were irrelevant in a discrimination learning procedure, by virtue of being nonpredictive and presented simultaneously with predictive cues, were compared with cues that were nonpredictive, but were not presented simultaneously with predictive cues (uncertain cues; Experiment 1). Our design was somewhat simpler than that used by Livesey et al. and we were interested in establishing the conditions under which changes in associability occur. Thus, task difficulty and complexity of the design of the task have been suggested as factors that may influence whether an uncertainty effect is observed (Le Pelley et al., 2016). In contrast to Livesey et al., who failed to find a difference between the two types of cues, we found that the irrelevant cues had lower associability than the uncertain cues. There are a number of potential explanations for this result, but the results of Experiments 2a and 2b demonstrated that the results of Experiment 1 reflected, at least in part, that uncertain cues increase in associability relative to other cues. Those results also contradicted the results of Livesey et al., who found the opposite effect: predictive cues were learned about more readily than uncertain cues. The subsequent experiments were devoted to identifying the key differences between our procedures and those used by Livesey et al. that determine whether predictiveness or uncertainty has the greatest effect on attention paid to a cue. Experiment 3 was a replication of the procedure used by Le Pelley and McLaren (2003) to determine whether we could replicate the predictiveness effect that they found using the stimuli that were used in Experiments 1 and 2a that produced the uncertainty effect. Experiment 4a and 4b used a more complex training procedure that involved a greater number of uncertain cues, which was similar to the procedure used by Livesey et al. In those experiments we found an effect of predictiveness rather than uncertainty. Experiment 5 replicated the procedures used in Experiment 2a and 2b (that produced an uncertainty effect) and Experiment 4b (that produced a predictiveness effect) with participants being assigned to one or the other procedure. Experiment 6 examined whether an increase in the number of cues was sufficient to result in a predictiveness effect. Finally, Experiments 7, 8a, and 8b examined whether the number of uncertain cues affected attention to cues that are both uncertain and relevant for discrimination learning by virtue of being part of a biconditional discrimination in which no one cue is informative, only the unique configurations of cues. This allowed assessment of the role of individual prediction error (for each cue, independent of other cues) and summed prediction error (across all cues present on a trial) in uncertainty and predictiveness effects on attention.

## Experiment 1

The purpose of Experiment 1 was to assess whether uncertainty, as determined by the summed associative strength of a compound of stimuli, affects the associability of cues. The design of Experiment 1 is shown in Table 1. In Stage 1, participants received trials with cues that were not predictive of outcomes by virtue of being paired equally often with two outcomes (i.e., Outcomes 1 and 2) across trials. Some of these nonpredictive cues were presented in compound with cues that were predictive across trials. Thus, Cues V through Y were nonpredictive, but were presented in compound with Cues A through D, which were predictive. For example, Cue V led to Outcome 1 when presented in compound with Cue A, but led to Outcome 2 when presented in compound with Cue B. In contrast, Cue A led to Outcome 1 regardless of the other cue in the compound (i.e., AV or AW). To differentiate between cues, we refer to the nonpredictive cues V through Y as *irrelevant cues*. Other nonpredictive cues were presented in compounds with cues that were also equally nonpredictive. Thus, Cues P through S were presented in the compounds PQ, RS, PS, and QR, and these compounds were paired with Outcomes 1 and 2 equally often. We refer to these nonpredictive cues as *uncertain cues*.[Table-anchor tbl1]

Although the uncertain and irrelevant cues have the same statistical relationship with Outcomes 1 and 2, the compounds in which they are presented differ in terms of their summed prediction error. The summed error reflects the discrepancy between the outcome and the combined predictive strength of all the cues present on a trial. The summed error of the compounds that consist of two uncertain cues will be high due to both cues being nonpredictive of the outcome. The summed error of the compounds that include the irrelevant cues will be lower, however, due to the presence of the predictive cues. Thus, as the associative strength of the predictive cue increases over the training, the summed error of the compound decreases. This is an important distinction given the assumptions of the Pearce–Hall model, which predicts that increases in attention to nonpredictive cues are driven by the summed error term rather than the individual error of cues. Therefore, the Pearce–Hall model anticipates that uncertain cues should receive more attention than irrelevant cues.

To assess whether uncertain cues gained more attention than irrelevant cues the associability of the stimuli was assessed in a second stage of training (see Table 1). Participants were presented with compounds consisting of one irrelevant cue and one uncertain cue. These new compounds were paired with new outcomes; either Outcome 3 or 4. Therefore, the irrelevant cues and uncertain cues were now equally predictive of these new outcomes. In the test phase participants were presented with novel compounds that consisted of either two irrelevant cues or two uncertain cues that had each led to the same outcome in Stage 2. Participants were asked to rate how likely Outcome 3 or 4 was given a particular compound. Greater attention to one type of cue over another would be indicated by more extreme ratings of the compounds for the correct outcome.

### Method

#### Participants

Twenty-four people (10 women, 14 men) participated in Experiment 1. The age range was 18 to 38 (*M* = 25.36, *SD* = 4.26). All participants had normal or corrected-to-normal vision. Durham University psychology undergraduates received participant-pool credit and others were compensated for their time at a rate of £10/hr ($13.07). All procedures were approved by the Department of Psychology Ethics Sub-Committee (15–10), Durham University.

The sample sizes across all experiments (except Experiment 3, see respective Method section) ranged from 21 to 32 (Experiment 5 used a between-subjects procedure with *n* = 24 per group). Variation between experiments reflected the number of participants that were available for testing within a particular time frame. For each experiment or between-subjects condition within an experiment, we aimed to test in excess of 20 participants similar to the study by Livesey et al. (2011) that used sample sizes of between 23 and 31 participants.

#### Apparatus and stimuli

All experimental stimuli were presented on a standard desktop computer with a 19-in. CRT monitor. Presentation of stimuli was controlled by MATLAB with CRS (Cambridge Research Systems, Rochester, England) toolbox and Psychtoolbox (Brainard, 1997). The distance between participants and the CRT monitor was 45 cm. Flags of the following countries were used as cues: United States, Brazil, Canada, China, United Kingdom, Spain, France, Germany, Israel, Japan, Korea, Mexico, Russia, Singapore, Sweden, Turkey, Benin, Guyana, Jamaica, The Republic of the Congo, Portugal, Cuba, Panama, and Uruguay. Each flag was 10° × 8° (Width × Length) in size. The outcomes (1 through 4) were represented by images depicting support (image of an apple), attack (image of a bomb), retreat (image of man running), and surrender (image of a man kneeling). Each outcome image was 4.6° × 4.3° in size. Participants made responses by clicking on a mouse.

#### Procedure

Participants were instructed that they would play the role of a soldier and were required to predict which outcome would be correct given the combination of flags presented. They were told that they would receive feedback for each choice, such that they could learn by trial and error as the procedure progressed. In Stage 1, each trial started with the presentation of two cues (flags) and two outcomes. Flags were presented in the top left and right corners of the screen. Outcomes were presented in the middle of the lower half of the screen. One outcome was presented above the other outcome. Participants had to choose to either select an upper outcome icon (e.g., bomb) or lower outcome icon (e.g., retreat) by using a left click of the mouse. Immediately after a response was made the word “Correct!” or “Incorrect” appeared in the center of the screen for one second. The next trial started immediately after the feedback screen. Participants received trials that belonged to one of two different conditions (see Table 1). In the predictive/irrelevant condition, participants were presented with pairs of flags. Across trials, individual cues would appear equally often with two other flags (e.g., on half the trials in which Cue A was presented, it would be presented with V and on the other half with W). The unique combination of flags on a particular trial always led to the same outcome in Stage 1, either Outcome 1 or 2. However, across trials, one flag in each compound was predictive in that it always led to the same outcome in Stage 1, independent of which flag it was paired with on a given trial (e.g., A was predictive of Outcome 1 when presented with other flags: AV→O1, AW→O1). The other flag in each compound was irrelevant in that it was not predictive by virtue of being paired with two different outcomes equally often (e.g., V was irrelevant when presented with other flags: AV→O1, BV→O2). Participants received eight trial types in the predictive/irrelevant condition: AV→O1, AW→O1, BV→O2, BW→O2, CX→O2, CY→O2, DX→O1, DY→O1. Cues A, B, C, and D were predictive, and V, W, X and Y were irrelevant. In the uncertain condition, participants were presented with pairs of flags that, across trials, led to two different outcomes equally often. Similar to the predictive/irrelevant condition, individual flags were each presented equally often with two other cues (e.g., PQ and PS), but independent of the particular compound that was presented, the probability of a particular outcome was 50%. Participants received four trial types in the uncertain condition: PQ→O1/O2, PS→ O1/O2, RQ→O1/O2, RS→O1/O2. Participants received 192 trials in total, with 16 trials of each trial type. The order of trial types across trials was random with the constraint that there was an equal number of each trial type every 48 trials. For every trial type, the spatial location of individual flags was balanced across every four trials of the same trial type so that each flag equally often occupied the left or right location (e.g., A on the left, V on the right; V on the left, A on the right). The spatial location (top or bottom) of Outcome 1 and 2 was random across trials.

In Stage 2, participants received eight trial types in which pairs of flags reliably led to either Outcome 3 or 4. Four of the eight trial types consisted of pairs of flags that included one irrelevant cue and one uncertain cue from Stage 1 (recombined cues: VP→O3, WQ→O4, XR→O3, YS→O4). For the remaining trial types, new flags that were not previously experienced in Stage 1 were used (EF→O3, GH→O4, IJ→O3, KL→O4). These trials with new flags were used as filler trials in order to increase the memory load of Stage 2, and replicated, in part, the procedure used by Le Pelley and McLaren (2003). Participants received 64 trials consisting of eight trials of each trial type. The order of trial types across trials was random with the constraint that there were an equal number of each trial type every 16 trials. All other details were the same as Stage 1.

In the test phase, participants were presented with novel pairings of the flags previously presented in Stage 2. Flags were presented in the top left and right corners of the screen in a similar manner to the previous training stages. Participants were asked to rate how likely Outcome 3 or Outcome 4 was given the combination of flags on a scale ranging from 1 to 9, which ran horizontally on the screen, with one outcome at one end of the scale and the other outcome at the other end. Participants were instructed that choosing either 1 or 9 would indicate that the outcome corresponding to the respective numbers was likely, whereas the other outcome was not. There were eight trial types. Two of the trial types consisted of pairs of flags that were previously irrelevant in Stage 1. One pair consisted of flags that had both led to Outcome 3 in Stage 2 (VX) and the other Outcome 4 (WY). Two of trial types consisted of pairs of flags that were previously uncertain in Stage 1. One pair consisted of flags that had both led to Outcome 3 in Stage 2 (PR) and the other Outcome 4 (QS). The remaining trial types consisted of the new flags presented in Stage 2. One trial type consisted of flags that led to Outcome 3 in Stage 2 (IJ), and another with flags that led to Outcome 4 (KL). The remaining trial types consisted of pairs of flags that had led to different outcomes during Stage 2 (EH and FG). The purpose of the test trials with the filler cues from Stage 2 was to test whether participants were able to use the rating scale appropriately and to replicate the procedure used by (Le Pelley & McLaren, 2003). Participants received two test trials with each trial type. The spatial location of each flag was balanced such that across trials each flag appeared equally often on the left and right. The location of Outcome 3 and 4 on the scale was random across trials.

The identity of each cue (A–D, P–S and V–Y) was random across participants. The identity of Outcomes 1 through 4 (apple, bomb, retreat, and surrender) was also random across participants.

#### Data analysis

The accuracy of responding, as measured by the proportion of correct responses for the different conditions was recorded during Stage 1 and 2 training. Performance was assessed over blocks of trials (the number of trials per block is stated in the relevant analyses). In the test stage the ratings were coded such that scores of 1 indicated that Outcome 3 was likely and scores of 9 indicated that Outcome 4 was likely. The mean score for the two test trials of each trial type was calculated. For all experiments, data were analyzed using multifactorial analyses of variance (ANOVAs). Interactions were analyzed by simple main effects analysis using the pooled error term from the original ANOVA.

Analysis of the filler trials was conducted in Stage 2 to determine whether these new cues were learned in addition to the recombined cues that were previously presented in Stage 2. For the sake of brevity, we have omitted analyses of the test trials with the filler cues; but for all experiments, performance on the filler cue test trials was as expected with ratings being below 5 for IJ and above 5 for KL, indicating that participants that learned the cue–outcome associations. Ratings for EH and FG were close to 5, consistent with the fact that each compound included one cue associated with Outcome 3 and another with Outcome 4.

For all experiments, no exclusion criteria were used and the initial data analysis was carried out on all participants. Other studies, such as Livesey et al. (2011), have used an exclusion criterion in order to eliminate participants that did not learn in the first stage of training. In order to aid comparison with studies that have employed an exclusion criterion, the number of subjects that failed to show performance of 60% and above in the last half of Stage 1 training on the soluble components of the learning task are reported. This criterion was used by Livesey et al. (2011). In addition, we report in the online supplemental material statistical analyses of the test phase on the subset of participants that met the criterion.

### Results and Discussion

#### Stage 1

Participants acquired the discrimination over training with performance increasing over blocks for the predictive/irrelevant condition, but no improvement for the uncertain condition. Mean performance on the last block (four trials of each trial type) was 84.24% (standard error of the mean [*SEM*] = 3.42) correct for the predictive/irrelevant condition and 46.61% (SEM = 2.19) for the uncertain condition. A repeated measures ANOVA with blocks (i.e., Blocks 1 through 4) and cue condition (predictive/irrelevant vs. uncertain) as factors showed significant main effects of both block, *F*(3, 69) = 12.85, *p* < .001, η_p_^2^ = .36, 90% CI [.19, .46], and cue condition, *F*(1, 23) = 74.82, *p* < .001, η_p_^2^ = .76, 90% CI [.58, .84], and a significant interaction between factors, *F*(3, 69) = 6.21, *p* = .001, η_p_^2^ = .21, 90% CI [.06, .32]. Two participants failed to show performance of 60% and above on the predictive/irrelevant condition in the last half of training. One of those participants failed to perform above 50% correct.

#### Stage 2

Participants acquired the discrimination over training. Learning was superior for the new cues compared with the recombined cues from Stage 1. Mean performance on the last block (two trials of each trial type) was 81.25% (*SEM* = 4.32) for the recombined condition and 92.71% (*SEM* = 2.59) for the novel condition. There was a significant effect of block, *F*(3, 69) = 35.50, *p* < .001, η_p_^2^ = .61, 90% CI [.47, .68], and cue condition, *F*(1, 23) = 18.50, *p* < .001, η_p_^2^ = .45, 90% CI [.18, .61], but no significant interaction of factors, *F*(3, 69) = 1.71, *p* = .17, η_p_^2^ = .07, 90% CI [.00, .15].

#### Test stage

**Figure 3 fig3:**
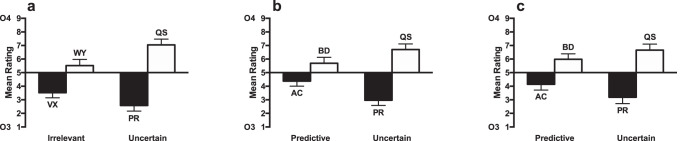
The results of the test phase of Experiments 1 (Panel a), 2a (Panel b), and 2b (Panel c). The likelihood that Outcomes 3 and 4 would occur for each test compound was rated on a scale ranging from 1 to 9 with scores below 5 indicating an expectation that Outcome 3 would occur and scores above 5, indicating that Outcome 4 would occur. Panel a: VX and WY were irrelevant cues paired with Outcomes 3 and 4, respectively. PR and QS were uncertain cues paired with Outcomes 3 and 4, respectively. Panel b and c: AC and BD were predictive cues paired with Outcomes 3 and 4, respectively. PR and QS were uncertain cues paired with outcomes 3 and 4, respectively. Error bars indicate standard error of the mean.

The ratings for the test stage are shown in Figure 3a. Participants rated the likelihood that Outcome 3 or Outcome 4 would occur on a nine-point scale. Scores below 5 indicated that participants expected Outcome 3, and scores above 5 indicated that participants expected Outcome 4. The raw ratings on the 1 to 9 score were analyzed. The ratings for compounds consisting of cues paired with Outcome 4 (WYand QS) were higher than for those paired with Outcome 3 (VX and PR), indicating that participants learned the cue–outcome associations. The difference between cues paired with Outcomes 3 and 4 was greater for the uncertain condition than the irrelevant condition. A 2 (cue condition: irrelevant cues [VX, WY] vs. uncertain cues [PR, QS]) × 2 (Outcome: 3 [VX, PR] vs. 4 [WY, QS]) ANOVA was conducted. There was a significant main effect of outcome, *F*(1, 23) = 29.11, *p* < .001, η_p_^2^ = .56, 90% CI [.30, .69], but no main effect of cue condition (*F* < 1, *p* = .43). There was an interaction between cue condition and outcome, *F*(1, 23) = 18.96, *p* < .001, η_p_^2^ = .45, 90% CI [.18, .61], demonstrating that the effect of outcome was significantly greater for the uncertain cues than for irrelevant cues. Simple main effects analysis of the interaction showed that there was a significant effect of outcome for the uncertain cues, *F*(1, 23) = 33.82, *p* < .001, η_p_^2^ = .60, 90% CI [.34, .72], and the irrelevant cues, *F*(1, 23) = 13.88, *p* = .001, η_p_^2^ = .37, 90% CI [.12, .55]. Analysis of the test phase results excluding the participants that failed to meet the Stage 1 learning criterion showed a similar pattern of results (see Table S1 and Figure S1a in the online supplemental material).[Fig-anchor fig3]

Following Stage 2 training, participants showed greater learning with the uncertain cues than with the irrelevant cues, indicating that, because of Stage 1 training, associability was greater for the uncertain cues than for the irrelevant cues. The results are not consistent with those reported by Livesey et al. (2011). They failed to find any difference between irrelevant and uncertain cues, and, therefore, concluded that attention was controlled by the individual prediction error for each cue rather than the summed error for each compound. Instead, our results are consistent with the prediction that uncertain cues receive greater attention than irrelevant cues because associability remains high due to the summed error calculated using the combined associative strength of both cues. For the irrelevant cues, the summed error per trial by the end of training was low for irrelevant cues, by virtue of participants learning about the predictive cues. In other words, the uncertain cues were able to benefit from increases in associability caused by a Pearce–Hall mechanism to a greater extent than the irrelevant cues (Haselgrove et al., 2010; Pearce & Hall, 1980). This possibility was explored in Experiments 2a and 2b.

In addition, whereas both our experiment and those of Livesey et al. (2011) tested changes in attention for irrelevant and uncertain cues, the procedure used in the current experiment differed from that used by Livesey et al. (2011) in a number of ways, which may have led to the difference in the results. The cause of the discrepancy between our findings and those of Livesey et al. (2011) were investigated in Experiments 2b, 4a, and 4b.

## Experiments 2a and 2b

In Experiment 1, uncertain cues were learned about more than were irrelevant cues. Irrelevant and uncertain cues were matched for individual prediction error because they had the same statistical relation with the outcomes. They differed, however, in the level of summed error. Although the summed error was high for the uncertain cues due to the associative strength of both cues in the compound being low, the summed error was low for the irrelevant cues because of the high associative strength of the predictive cues.

Although it is possible that the results of Experiment 1 demonstrate an effect of uncertainty on attention, as determined by the summed error of the compound, there are other differences between the conditions (uncertain/irrelevant) which may have contributed to the observed difference. One possibility is that, as a specific consequence of the predictive cues, participants learned to ignore the irrelevant cues. This learned response to the irrelevant cues may have then carried over to training in Stage 2 such that participants attended to uncertain cues rather than irrelevant cues. If this was the case, then there is no need to assume that the associability of uncertain cues increased because of Stage 1 training. Indeed, it would be expected that uncertain cues would receive less attention than predictive cues.

The purpose of Experiment 2a was to test whether uncertain cues receive more attention than predictive cues because of Stage 1 training. Such evidence, combined with the results of Experiment 1, would suggest that uncertain cues undergo an increase in associability relative to predictive/irrelevant cues due the summed error of the compound. The procedure for Experiment 2a was similar to that for Experiment 1, except in Stage 2 participants were presented with compounds that consisted of one predictive cue and one uncertain cue (see Table 1). In the test phase, participants were required to rate how likely Outcomes 3 or 4 were given compounds that consisted of either predictive cues or uncertain cues that had both led to the same outcome in Stage 2.

Experiment 2b was a replication of 2a except that the stimuli were the same as those used by Livesey et al. (2011). This was done to rule out the possibility that choice of stimuli and cover story influenced the likelihood of observing an uncertainty effect.

### Method

#### Participants

Thirty-two people (24 women, eight men) participated in Experiment 2a. The age range was 18 to 32 (*M* = 23.63, *SD* = 4.25). Thirty-two participants (20 women, 12 men) participated in Experiment 2b. The age range was 20 to 32 (*M* = 25.59, *SD =* 3.63). All other details were the same as Experiment 1.

#### Apparatus and stimuli

For Experiment 2a, all apparatus and stimuli were the same as Experiment 1. For Experiment 2b, all details were the same as Experiment 2a, except the stimuli used for cues and objects were the same as used by Livesey et al. (2011). Specifically, the cue images were line drawings of familiar objects taken from the Snodgrass and Vanderwart (1980) standardized set of pictures (4.6° × 4.3°). The images used for outcomes were rain, snow, hail, and fog.

#### Procedure

For Experiment 2a, the procedure for Stage 1 of training was the same as Experiment 1 (see Table 1). Stage 2 training was similar to Experiment 1 but now recombined compounds each consisted of one uncertain flag and one predictive flag (AP→O3, BQ→O4, CR→O3, DS→O4). The test stage proceeded in a similar manner to Experiment 1. In addition to testing with the novel flags presented in Stage 2 (test compounds: EH, FG, IJ, KL), participants were tested with pairs of flags that both led to the same outcome in Stage 2 and were both either uncertain cues (PR, QS) or predictive cues (AC, BD) in Stage 1. For Experiment 2b, the procedure was the same as Experiment 2a except that participants were given instructions similar to those used by Livesey et al. (2011), in which they were told to use the line drawing images in order to predict the weather (i.e., which weather outcome will occur: rain, snow, hail, and fog).

### Results

#### Experiment 2a

##### Stage 1

Participants acquired the discrimination over training with performance increasing for the predictive/irrelevant condition over blocks, but no improvement for the uncertain condition. Mean performance on the last block (four trials of each trial type) was 76.56% (*SEM* = 3.48) for the predictive/irrelevant condition and 47.27% (*SEM* = 2.21) for the uncertain condition. There was a significant effect of block, *F*(3, 93) = 10.64, *p* < .001, η_p_^2^ = .26, 90% CI [.12, .35], and condition, *F*(1, 31) = 41.48, *p* < .001, η_p_^2^ = .57, 90% CI [.36, .69], and a significant interaction between factors, *F*(3, 93) = 6.34, *p* = .001, η_p_^2^ = .17, 90% CI [.05, .26]. Seven participants failed to show performance of 60% and above on the predictive/irrelevant condition in the last half of training. Four of those participants failed to perform above 50% correct.

##### Stage 2

Participants acquired the discrimination over training for both the novel cues and the recombined cues from Stage 1. Mean performance on the last block (two trials of each trial type) was 81.25% (*SEM* = 3.81) for the recombined condition and 83.20% (*SEM* = 3.05) for the novel condition. There was a significant effect of block, *F*(3, 93) = 33.67, *p* < .001, η_p_^2^ = .52, 90% CI [.39, .60], but no significant main effect of condition, *F*(1, 31) = 2.41, *p* = .13, η_p_^2^ = .07, 90% CI [.00, .24]. There was no significant interaction of factors, *F* < 1, *p* = .41.

##### Test stage

The ratings for the test stage are shown in Figure 3b. The ratings for compounds consisting of cues paired with Outcome 4 (BD, QS) were higher than for those paired with Outcome 3 (AC, PR), indicating that participants learned the cue–outcome associations. The difference between cues paired with Outcomes 3 and 4 was greater for the uncertain condition than for the predictive condition. A 2 (predictiveness: predictive cues [AC, BD] vs. uncertain cues [PR, QS]) × 2 (Outcome: 3 [AC, PR] vs. 4 [BD, QS]) ANOVA was conducted. There was a significant main effect on outcome, *F*(1, 31) = 21.23, *p* < .001, η_p_^2^ = .41, 90% CI [.18, .56], but no main effect on predictiveness (*F* < 1, *p* = .42). There was an interaction between predictiveness and outcome, *F*(1, 31) = 11.47, *p* = .002, η_p_^2^ = .27, 90% CI [.07, .45], demonstrating that the effect of outcome was significantly greater for the uncertain cues than for predictive cues. Simple main effects analysis of the interaction showed that there was a significant effect of outcome for the uncertain cues, *F*(1, 31) = 31.59, *p* < .001, η_p_^2^ = .50, 90% CI [.28, .64]. The effect for the predictive cues narrowly failed to reach significance, *F*(1, 31) = 4.16, *p* = .05, η_p_^2^ = .12, 90% CI [.00, .30]. Analysis of the test phase results excluding the participants that failed to meet the Stage 1 learning criterion showed a similar pattern of results (see Table S1 and Figure S1b in the online supplemental material).

#### Experiment 2b

##### Stage 1

Participants acquired the discrimination over training with performance increasing for the predictive/irrelevant condition over blocks, but no improvement for the uncertain condition. Mean performance on the last block (four trials of each trial type) was 78.13% (*SEM* = 3.31) for the predictive/irrelevant condition and 45.70% (*SEM* = 2.20) for the uncertain condition. There was a significant effect of block, *F*(3, 93) = 6.60, *p* < .001, η_p_^2^ = .18, 90% CI [.06, .27], and condition, *F*(1, 31) = 93.34, *p* < .001, η_p_^2^ = .75, 90% CI [.60, .82], and a significant interaction between factors, *F*(3, 93) = 6.94, *p* = .001, η_p_^2^ = .18, 90% CI [.06, .28]. Eight participants failed to show performance of 60% and above on the predictive/irrelevant condition in the last half of training. One of those participants failed to perform above 50% correct.

##### Stage 2

Participants acquired the discrimination over training for both the novel cues and the recombined cues from Stage 1. Mean performance on the last block (two trials of each trial type) was 86.72% (*SEM* = 2.69) for the recombined condition and 86.72% (*SEM* = 3.50) for the novel condition. There was a significant effect of block, *F*(3, 93) = 41.77, *p* < .001, η_p_^2^ = .57, 90% CI [.45, .64], but no significant main effect of condition (*F* < 1, *p* = .69). There was no significant interaction of factors, *F*(3, 93) = 2.19, *p* = .10, η_p_^2^ = .07, 90% CI [.00, .14].

##### Test stage

The ratings for the test stage are shown in Figure 3c. The ratings for compounds consisting of cues paired with Outcome 4 (BD, QS) were higher than for those paired with Outcome 3 (AC, PR), indicating that participants learned the cue–outcome associations. The difference between cues paired with Outcomes 3 and 4 was greater for the uncertain condition than the predictive condition. A 2 (predictiveness: predictive cues [AC, BD] vs. uncertain cues [PR, QS]) × 2 (Outcome: 3 [AC, PR] vs. 4 [BD, QS]) ANOVA was conducted. There was a significant main effect on outcome, *F*(1, 31) = 16.22, *p* < .001, η_p_^2^ = .34, 90% CI [.12, .51], but no main effect on predictiveness (*F* < 1, *p* = .59). The interaction narrowly failed to reach significance, *F*(1, 31) = 4.15, *p* = .050, η_p_^2^ = .12, 90% CI [.00, .30]. Simple main effects analysis showed that the effect of outcome was significant for both the uncertain, *F*(1, 31) = 19.18, *p* < .001, η_p_^2^ = .38, 90% CI [.16, .54], and predictive cues, *F*(1, 31) = 6.06, *p* = .02, η_p_^2^ = .16, 90% CI [.01, .34]. Analysis of the test phase results excluding the participants that failed to meet the Stage 1 learning criterion showed a similar pattern of results (see Table S1 and Figure S1c in the online supplemental material).

Although the interaction between predictiveness and outcome failed to reach the threshold for significance (*p* = .05 rather than <0.05), it was clear that the pattern of results was similar to those in Experiment 2a, suggesting that uncertain cues received more attention than predictive cues. To calculate the strength of evidence for replication of the results of Experiment 2a, we used the procedure proposed by Ly, Etz, Marsman, and Wagenmakers (2019) for calculating a replication Bayes factor (BF): The BF for the two experiments combined as a ratio of the BF for Experiment 2a. We conducted Bayesian *t* tests comparing the difference between QS and PR with the difference between BD and AC using the JASP software (Love et al., 2019). For Experiment 2a, BF_10_ = 18.21, and for both experiments combined BF_10_ = 69.74. Therefore, the replication BF_10_ = 3.83 suggests that the evidence for replication of the uncertainty effect was 3.83 times stronger than that for no effect of uncertainty.

The results of the test phase for both Experiments 2a and 2b suggest that participants paid more attention to uncertain cues than to predictive cues. They rule out the possibility from Experiment 1 that the greater attention paid to uncertain cues in that experiment was simply the result of participants ignoring irrelevant cues, because the comparison in Experiments 2a and 2b was between uncertain cues and predictive cues. These results are in line with the predictions of the Pearce and Hall (1980) model in which attention is positively related to size of prediction error based on a summed error term. The results contradict the prediction of Mackintosh (1975) that attention is inversely related to prediction error based on the individual error term for each cue independent of the associative strength of other cues.

Once again, the results do not match those of Livesey et al. (2011). In that study, it was found that uncertain cues had lower associability than predictive cues. The opposite effect was observed in Experiments 2a and an increase in associability of uncertain cues relative to predictive cues was replicated using the same stimuli as Livesey et al. (2011) in Experiment 2b.

## Experiment 3

Experiments 1 and 2 demonstrate that uncertain cues had greater associability than both irrelevant cues and predictive cues. The results contrast with a large number of experiments that have demonstrated that nonpredictive cues have lower associability than predictive cues (Le Pelley et al., 2016). This finding has been most commonly demonstrated when predictive cues have competed with irrelevant cues for attention (see Le Pelley & McLaren, 2003 for a review; Le Pelley et al., 2016). Given that the results of Experiments 1 and 2 contradict the findings of other studies it was important to establish that we could replicate the finding that predictive cues receive more attention than irrelevant cues using procedures similar to Experiments 1 and 2. If this could not be replicated it may suggest that there was something specific about the procedures of Experiments 1 and 2 that had led to uncertain cues receiving more attention than predictive and irrelevant cues. Therefore, in Experiment 3 it was assessed whether predictive cues receive more attention than irrelevant cues. In contrast to Experiments 1 and 2, participants received training with only compounds of predictive and irrelevant cues in Stage 1 and did not receive training with uncertain compounds (see Table 2). This resulted in the procedure being similar to that used by Le Pelley and McLaren (2003).[Table-anchor tbl2]

### Method

#### Participants

Sixteen people (12 women, four men) took part in the experiment. The age range was 18 to 31 (*M* = 23.7, *SD* = 4.5). All other details were the same as Experiment 1. Based on a power analysis of the results of Le Pelley and McLaren (2003), a sample size of 16 was deemed sufficient to achieve power in excess of 0.8 (α = .05).

#### Apparatus and stimuli

All details were the same as Experiment 1.

#### Procedure

In Stage 1 participants received training with eight compounds that were presented in the same manner as the predictive/irrelevant condition in Experiment 1, in which one cue in each compound was predictive over trials, and the other cue was irrelevant by virtue of being paired equally often with Outcomes 1 and 2 across trials (see Table 2). Participants received no other trial types during Stage 1. In contrast to Experiment 1, participants received 14 rather 16 presentations of each trial type, matching the Stage 1 training procedure used by Le Pelley and McLaren (2003). All other details were the same as Experiment 1.

In Stage 2, participants received trials in which the cues from Stage 1 were recombined in new compounds. These compounds were then paired with either Outcome 3 or 4. Each compound consisted of one predictive cue from Stage 1 and one irrelevant cue from Stage 1. Compounds AX and CV were paired with Outcome 3 and BY and DW were paired with Outcome 4. In the test stage, participants were presented with compounds that consisted of either predictive cues (AC, BD) or irrelevant cues (VX, WY). Compounds AC and VX consisted of cues previously paired with Outcome 3 in Stage 2 and compounds BD and WY consisted of cues previously paired with Outcome 4 in Stage 2. All other details for Stage 2 and the test phase were the same as Experiment 1.

### Results and Discussion

#### Stage 1

Participants acquired the discrimination over training with performance increasing over blocks. Mean performance on the last block (two trials of each trial type) was 89.45% (*SEM* = 3.31). There was a significant effect of block, *F*(6, 90) = 6.56, *p* = .008, η_p_^2^ = .30, 90% CI [.14, .38]. One participant failed to show performance of 60% or above across the last three blocks of training (six trials of each trial type). Their performance was 56.25% correct.

#### Stage 2

Participants acquired the discrimination over training for both the novel cues and the recombined cues from Stage 1. Performance on the last block (two trials of each trial type) was 90.63% (*SEM* = 4.91) for the recombined condition and 93.75% (*SEM* = 2.28) for the novel condition. There was a significant effect of block, *F*(3, 45) = 86.23, *p* < .001, η_p_^2^ = .85, 90% CI [.77, .88], but no significant effect of condition (*F* < 1, *p* = .65) or interaction of factors, *F*(3, 45) = 1.10, *p* = .36, η_p_^2^ = .07, 90% CI [.00, .16].

#### Test

**Figure 4 fig4:**
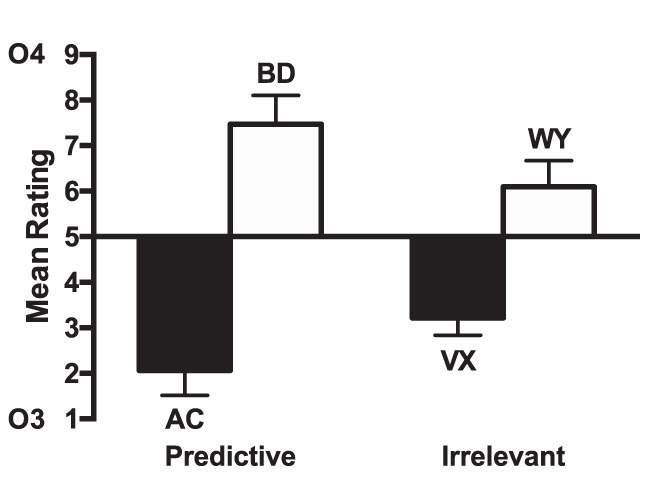
The results of the test phase of Experiment 3. The likelihood that Outcomes 3 and 4 would occur for each test compound was rated on a scale ranging from 1 to 9, with scores below 5 indicating an expectation that Outcome 3 would occur and scores above 5 indicating that Outcome 4 would occur. AC and BD were predictive cues paired with Outcomes 3 and 4, respectively. VX and WY were irrelevant cues paired with Outcomes 3 and 4, respectively. Error bars indicate standard error of the mean.

The ratings for the test stage are shown in Figure 4. The ratings for compounds consisting of cues paired with Outcome 4 (BD, WY) were higher than for those paired with Outcome 3 (AC, VX), indicating that participants learned the cue–outcome associations. The difference between cues paired with Outcomes 3 and 4 was greater for the predictive condition than the irrelevant condition. A 2 (predictiveness: predictive cues [AC, BD] vs. irrelevant [VX, WY]) × 2 (Outcome: 3 [AC, VX] vs. 4 [BD, WY]) ANOVA was conducted. There was a significant main effect on outcome, *F*(1, 15) = 32.08, *p* < .001, η_p_^2^ = .68, 90% CI [.38, .79], but no main effect of predictiveness, *F* < 1, *p* = .7. There was a significant predictiveness by outcome interaction, *F*(1, 15) = 9.45, *p* = .008, η_p_^2^ = .39, 90% CI [.07, .59], indicating that the effect of outcome was significantly greater for the predictive condition, *F*(1, 15) = 22.80, *p* < .001, η_p_^2^ = .60, 90% CI [.28, .74], than the irrelevant condition, *F*(1, 15) = 15.23, *p* = .001, η_p_^2^ = .50, 90% CI [.17, .67]. Analysis of the test phase results excluding the participants that failed to meet the Stage 1 learning criterion showed a similar pattern of results (see Table S1 and Figure S2 in the online supplemental material).[Fig-anchor fig4]

The results of the test stage demonstrate that predictive cues were learned to a greater extent than irrelevant cues in Stage 2, replicating the results of other experiments that have used similar experimental designs (e.g., Le Pelley & McLaren, 2003). Therefore, it was still possible to demonstrate a role for predictiveness in attention using the same stimuli and cover story used in Experiments 1 and 2. This suggests that, because Livesey et al. (2011) found that uncertain cues received less attention than predictive cues, the results of Experiments 2a and 2b, in which we found the opposite result, were likely to be due to a specific aspect of the experimental design.

## Experiments 4a and 4b

Other than the nature of the stimuli, Experiment 2a and 2b differed from the experiments reported by Livesey et al. (2011) in the general complexity of the training procedures. In the study by Livesey et al. (2011) participants were exposed to eight uncertain cues across eight compounds, resulting in the number of trial types that were impossible to learn being higher than in Experiment 2a and 2b, in which there were only four uncertain compounds. Therefore, to test whether the number of uncertain compounds determined the effects of predictiveness and uncertainty the number of uncertain compounds was increased from four to eight in Experiments 4a and 4b.

Experiments 4a and 4b were identical except for the combination of stimuli that were used in Stage 2 and the test phase (see Table 3). In Experiment 4a uncertain cues were combined in the test phase in a manner that was similar to that used by Livesey et al. (2011). In Experiment 4b uncertain cues were combined in the test phase in the same manner as that used in Experiments 2a and 2b.[Table-anchor tbl3]

### Method

#### Participants

Twenty-four people (15 women, 9 men) participated in Experiment 4a. The age range was 18 to 31 (*M* = 22, *SD =* 3.74). Twenty-six people (24 women, 2 men) participated in Experiment 4b. The age range was 18 to 28 (*M* = 20.6, *SD* = 3.13). All other details were the same as Experiment 1.

#### Apparatus and stimuli

For both Experiment 4a and 4b, all details were the same as Experiment 2a.

#### Procedure

The details of Stage 1 training were the same for Experiment 4a and 4b (see Table 3). Stage 1 training was the same as Experiments 2a and 2b except participants now received additional training with four extra uncertain compounds (ZM, ZO, NO, NM). These compounds were presented in the same manner as the other uncertain compounds (PQ, PS, RS, RQ). For Experiment 4a, in Stage 2 participants received training that was similar to Experiments 2a and 2b except that the recombined compounds were now AP, BR, CZ, and DN. AP and CZ led to Outcome 3 and BR and DN led to Outcome 4. The test phase was similar to Experiments 2a and 2b except that participants were tested with the compounds AC, BD, PZ, and NR in addition to the control compounds EH, FG, IJ, and KL. Similar to Experiments 2a and 2b, compounds AC and BD consisted of predictive cues from Stage 1 and were paired with Outcomes 3 and 4, respectively, in Stage 2. Compounds PZ and NR consisted of uncertain cues from Stage 1 and were paired with Outcomes 3 and 4, respectively, in Stage 2. The details of Stage 2 training and the test phase for Experiment 4b were the same as Experiments 2a and 2b.

### Results

#### Experiment 4a

##### Stage 1

Participants acquired the discrimination over training with performance increasing for the predictive/irrelevant condition over blocks, but no improvement for the uncertain condition. Mean performance on the last block (four trials of each trial type) was 66.80% (*SEM* = 3.19) for the predictive/irrelevant condition and 50.26% (*SEM* = 1.87) for the uncertain condition. There was a significant effect of block, *F*(3, 69) = 8.52, *p* < .001, η_p_^2^ = .27, 90% CI [.11, .38], and condition, *F*(1, 23) = 30.37, *p* < .001, η_p_^2^ = .57, 90% CI [.31, .70], but no significant interaction between factors, *F*(3, 69) = 1.48, *p* = .23, η_p_^2^ = .06, 90% CI [.00, .14]. Seven participants failed to show performance of 60% and above on the predictive/irrelevant condition in the last half of training. Three of those participants failed to show performance above 50% correct.

##### Stage 2

Participants acquired the discrimination over training for both the novel cues and the recombined cues from Stage 1. Mean performance on the last block (two trials of each trial type) was 84.90% (*SEM* = 3.28) for the recombined condition and 88.02% (*SEM* = 2.55) for the novel condition. There was a significant effect of block, *F*(3, 69) = 26.64, *p* < .001, η_p_^2^ = .54, 90% CI [.38, .62], and a significant main effect of condition, *F*(1, 23) = 4.54, *p* = .045, η_p_^2^ = .16, 90% CI [.003, .37]. There was no significant interaction of factors, *F*(3, 69) = 1.21, *p* = .31, η_p_^2^ = .05, 90% CI [.00, .12].

##### Test stage

**Figure 5 fig5:**
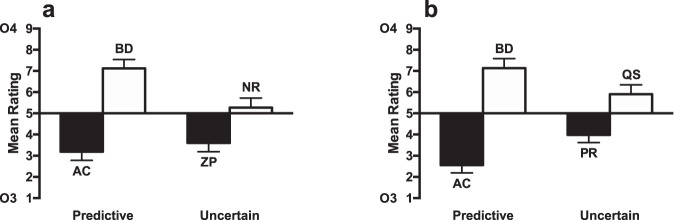
The results of the test phase of Experiments 4a (Panel a) and 4b (Panel B). The likelihood that Outcomes 3 and 4 would occur for each test compound was rated on a scale ranging from 1 to 9, with scores below 5 indicating an expectation that Outcome 3 would occur and scores above 5 indicating that Outcome 4 would occur. AC and BD were predictive cues paired with Outcomes 3 and 4, respectively. In Experiment 5 (Panel a), ZP and NR were uncertain cues paired with Outcomes 3 and 4, respectively. In Experiment 6 (Panel b), PR and QS were uncertain cues paired with Outcomes 3 and 4, respectively. Error bars indicate standard error of the mean.

The ratings for the test stage are shown in Figure 5a. The ratings for compounds consisting of cues paired with Outcome 4 (BD, NR) were higher than for those paired with Outcome 3 (AC, PZ), indicating that participants learned the cue–outcome associations. The difference between cues paired with Outcomes 3 and 4 was greater for the predictive condition than the uncertain condition. A 2 (predictiveness: predictive cues [AC, BD] vs. uncertain cues [PZ, NR]) × 2 (Outcome: 3 [AC, PZ] vs. 4 [BD, NR]) ANOVA was conducted. There was a significant main effect of outcome, *F*(1, 23) = 41.53, *p* < .001, η_p_^2^ = .64, 90% CI [.41, .75]. The effect of predictiveness failed to reach significance, *F*(1, 23) = 3.62, *p* = .07, η_p_^2^ = .14, 90% CI [.00, .34]. The interaction between these factors was significant, *F*(1, 23) = 13.85, *p* = .001, η_p_^2^ = .38, 90% CI [.12, .55], indicating that the effect of outcome was significantly greater for the predictive condition than the uncertain condition. Simple main effects analysis showed that the effect of outcome was significant for both the uncertain, *F*(1, 23) = 12.04, *p* = .002, η_p_^2^ = .34, 90% CI [.09, .52], and predictive cues, *F*(1, 23) = 46.47, *p* < .001, η_p_^2^ = .67, 90% CI [.44, .77]. Analysis of the test phase results excluding the participants that failed to meet the Stage 1 learning criterion showed a similar pattern of results (see Table S1 and Figure S3a in the online supplemental material).[Fig-anchor fig5]

#### Experiment 4b

##### Stage 1

Participants acquired the discrimination over training with performance increasing for the predictive/irrelevant condition over blocks, but no improvement for the uncertain condition. Mean performance on the last block (four trials of each trial type) was 71.03% (*SEM* = 3.63) for the predictive/irrelevant condition and 47.72% (*SEM* = 1.62) for the uncertain condition. There was no significant effect of block, *F*(3, 75) = 2.46, *p* < .07, η_p_^2^ = .09, 90% CI [.00, .17], but there was a significant effect of condition, *F*(1, 25) = 29.68, *p* < .001, η_p_^2^ = .54, 90% CI [.29, .67], but no significant interaction between factors, *F*(3, 75) = 1.55, *p* = .21, η_p_^2^ = .06, 90% CI [.00, .13]. Nine participants failed to show performance of 60% and above on the predictive/irrelevant condition in the last half of training. Six of those participants failed to perform above 50% correct.

##### Stage 2

Participants acquired the discrimination over training for both the novel cues and the recombined cues from Stage 1. Mean performance on the last block (two trials of each trial type) was 82.69% (*SEM* = 3.33) for the recombined condition and 89.90% (*SEM* = 2.78) for the novel condition. There was a significant effect of block, *F*(3, 75) = 22.78, *p* < .001, η_p_^2^ = .48, 90% CI [.32, .56], and a significant main effect of condition, *F*(1, 25) = 8.66, *p* = .007, η_p_^2^ = .26, 90% CI [.05, .45]. There was no significant interaction of factors, *F* < 1, *p* = .94.

##### Test stage

The ratings for the test stage are shown in Figure 5b. The ratings for compounds consisting of cues paired with Outcome 4 (BD, QS) were higher than for those paired with Outcome 3 (AC, PR), indicating that participants learned the cue–outcome associations. The difference between cues paired with Outcomes 3 and 4 was greater for the predictive condition than the uncertain condition. A 2 (predictiveness: predictive cues [AC, BD] vs. uncertain cues [PR, QS]) × 2 (Outcome: 3 [AC, PR] vs. 4 [BD, QS]) ANOVA was conducted. There was a significant main effect on outcome, *F*(1, 25) = 42.12, *p* < .001, η_p_^2^ = .63, 90% CI [.40, .74], but no significant effect of predictiveness, *F* < 1, *p* = .76. The interaction between these factors was significant, *F*(1, 25) = 7.23, *p* = .013, η_p_^2^ = .22, 90% CI [.03, .42], indicating that the effect of outcome was significantly greater for the predictive condition than the uncertain condition. Simple main effects analysis showed that the effect of outcome was significant for both the uncertain, *F*(1, 25) = 8.81, *p* = .007, η_p_^2^ = .26, 90% CI [.05, .45], and predictive cues, *F*(1, 25) = 36.82, *p* < .001, η_p_^2^ = .60, 90% CI [.36, .71]. Analysis of the test phase results excluding the participants that failed to meet the Stage 1 learning criterion showed a similar pattern of results (see Table S1 and Figure S3b in the online supplemental material).

To calculate the strength of evidence for replication of the results of Experiment 4a we calculated a replication BF (the BF for the two experiments combined as a ratio of the BF for Experiment 4a). We conducted Bayesian *t* tests comparing the difference between QS and PR with the difference between BD and AC. For Experiment 4a, BF_10_ = 31.77 and for both experiments combined, BF_10_ = 216.7. Therefore, the replication BF_10_ = 6.82 suggests that evidence for replication of the predictiveness effect was 6.82 times greater than for no replication of the predictiveness effect.

### Discussion

Experiments 4a and 4b demonstrated that predictive cues were learned more readily than uncertain cues in Stage 2 suggesting that predictive cues received more attention than uncertain cues. These results are consistent with the those of Livesey et al. (2011), but are inconsistent with the results of Experiments 2a and 2b, in which fewer uncertain cues were learned in Stage 1 training. This suggests that a factor that determines whether attention is paid to uncertain cues or predictive cues is the number of uncertain cues in Stage 1 training. This factor was tested directly in Experiment 5.

## Experiment 5

### Method

The procedures for Experiments 2a, 2b, and 4b were identical except for the number of uncertain compounds in Stage 1. When participants had few uncertain compounds in Stage 1 they learned more about uncertain cues than predictive cues (Experiment 2a and 2b), but when they received a greater number of uncertain compounds they learned more about predictive cues than uncertain cues (Experiment 4b). Experiment 5 directly tested the effect of the number of uncertain compounds on changes in associability of predictive and uncertain cues. Participants were trained with either four uncertain compounds in Stage 1 (replicating the procedure of Experiment 2a and 2b) or with eight uncertain compounds (replicating the procedure of Experiment 4a and 4b).

#### Participants

Forty-eight people participated in the experiment and were randomly allocated to one of two groups (*n* = 24 per group). In group few, there were 17 women and seven men, and the age range was 18 to 31 (*M* = 23.96, *SD* = 6.00). In group many, there were 15 women and nine men, and the age range was 19 to 35 (*M* = 22.79, *SD*: 4.05). All participants had normal or corrected to normal vision. All other details were the same as Experiment 1.

#### Apparatus and Stimuli

All details were the same as Experiments 2a and 4b.

#### Procedure

Participants were randomly allocated to one of two groups. Group few received the same procedure as Experiment 2a in which there were four uncertain compounds in Stage 1. Group many received the same procedure as Experiment 4b in which there eight uncertain compounds. The procedures of Experiment 2a and 4b, and therefore, also of groups few and many were identical in all respects except in the number of uncertain compounds in Stage 1 (see Tables 1 and 3).

### Results and Discussion

#### Stage 1

Participants acquired the discrimination over training with performance increasing for the predictive/irrelevant condition over blocks, but no improvement for the uncertain condition. Mean performance on the last block (four trials of each trial type) for the predictive/irrelevant condition was 77.08% (*SEM* = 3.25) for group few and 70.31% (*SEM* = 3.21) for group many. For the uncertain condition, it was 47.40% (*SEM* = 1.80) for group few and 45.96% (*SEM* = 1.49) for group many. There was a significant effect of predictiveness, *F*(1, 46) = 87.59, *p* < .001, η_p_^2^ = .66, 90% CI [.51, .74], which interacted with block, *F*(3, 138) = 16.65, *p* < .001, η_p_^2^ = .27, 90% CI [.15, .35]. Block also interacted with group, *F*(3, 138) = 2.79, *p* = .043, η_p_^2^ = .06, 90% CI [.001, .11], but there was no significant predictiveness by group interaction, *F*(1, 46) = 2.21, *p* = .14, η_p_^2^ = .05, 90% CI [.00, .17], nor significant predictiveness by group by block three-way interaction, *F* < 1, *p* = .53, suggesting that any difference between the two groups over the course of training was not specific to the predictive/irrelevant condition. Five participants in group few and seven in group many failed to show performance of 60% and above on the predictive/irrelevant condition in the last half of training. Of those participants, one in group few and one in group many failed to perform above 50% correct.

#### Stage 2

Participants acquired the discrimination over training for both the novel cues and the recombined cues from Stage 1. Mean performance on the last block (two trials of each trial type) for the recombined condition was 81.77% (*SEM* = 4.70) for group few and 80.21% (*SEM* = 3.60) for group many. For the novel condition is was 86.46% (*SEM* = 3.09) for group few and 83.85% (*SEM* = 3.49) for group many. There was a significant effect of block, *F*(3, 138) = 52.60, *p* < .001, η_p_^2^ = .53, 90% CI [.43, .60], but no other significant effects or interactions, *F*s < 1.6, *p*s > 0.2.

#### Test stage

**Figure 6 fig6:**
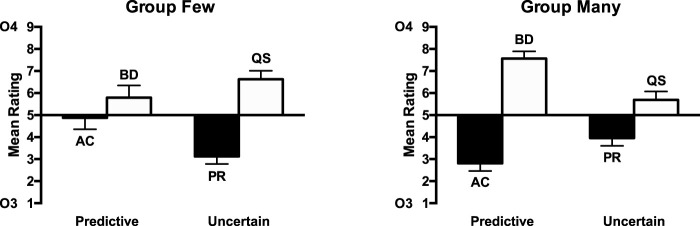
The results of the test phase of Experiment 5. The mean ratings for group few are on the left and those for group many are on the right. The likelihood that Outcomes 3 and 4 would occur for each test compound was rated on a scale ranging from 1 to 9, with scores below 5 indicating an expectation that Outcome 3 would occur and scores above 5 indicating that Outcome 4 would occur. AC and BD were predictive cues paired with Outcomes 3 and 4, respectively. PR and QS were uncertain cues paired with Outcomes 3 and 4, respectively. Error bars indicate standard error of the mean.

The ratings for the test stage are shown in Figure 6. The ratings for compounds consisting of cues paired with Outcome 4 (BD, QS) were higher than for those paired with Outcome 3 (AC, PR), indicating that participants learned the cue–outcome associations. For group few, the difference between cues paired with Outcomes 3 and 4 was greater for the uncertain condition than the predictive condition. The opposite was true for group many, with the difference between the cues paired with Outcomes 3 and 4 being greater for the predictive cues than for the uncertain cues. A 2(group: few vs. many) × 2 (predictiveness: predictive cues [AC, BD] vs. uncertain cues [PR, QS]) × 2 (Outcome: 3 [AC, PR] vs. 4 [BD, QS]) ANOVA was conducted. There was a significant effect of outcome, *F*(1, 46) = 56.25, *p* < .001, η_p_^2^ = .55, 90% CI [.38, .66], but no significant effect of predictiveness, *F*(1, 46) = 2.73, *p* = .11, η_p_^2^ = .06, 90% CI [.00, .19]. There was no significant interaction between predictiveness and outcome, *F* < 1, *p* = .70, but there was a significant three-way interaction between group, predictiveness and outcome, *F*(1, 46) = 24.50, *p* < .001, η_p_^2^ = .35, 90% CI [.16, .49]. There were no other significant effects or interactions of factors, *p*s > 0.1. The three-way interaction was explored by conducting separate ANOVAs for each group. For group few there was a significant predictiveness by outcome interaction, *F*(1, 23) = 8.21, *p* = .009, η_p_^2^ = .26, 90% CI [.04, .46], indicating that the effect of outcome was significantly greater for the uncertain condition than the predictive condition. Simple main effects analysis showed that though there was a significant effect of outcome for the uncertain cues, *F*(1, 23) = 29.66, *p* < .001, η_p_^2^ = .56, 90% CI [.30, .69], but there was not for the predictive cues, *F*(1, 23) = 1.23, *p* = .28, η_p_^2^ = .05, 90% CI [.00, .23]. For group many, there was a significant predictiveness by outcome interaction, *F*(1, 23) = 19.47, *p* < .001, η_p_^2^ = .46, 90% CI [.19, .62], indicating that the effect of outcome was significantly greater for the predictive condition than for the uncertain condition. Simple main effects analysis showed that the effect of outcome was significant for both predictive, *F*(1, 23) = 77.12, *p* < .001, η_p_^2^ = .77, 90% CI [.59, .84], and uncertain cues, *F*(1, 23) = 9.71, *p* = .005, η_p_^2^ = .30, 90% CI [.06, .49]. Analysis of the test phase results excluding the participants that failed to meet the Stage 1 learning criterion showed a similar pattern of results (see Table S1 and Figure S4 in the online supplemental material).[Fig-anchor fig6]

The results of group few replicated the uncertainty effect observed in Experiments 2a and 2b and the results of group many replicated the predictiveness effect observed in Experiments 4a and 4b. To calculate the strength of evidence for replication of the uncertainty and predictiveness effects we calculated replication BFs. We conducted Bayesian *t* tests comparing the difference between QS and PR with the difference between BD and AC. For the uncertainty effect observed in group few, the replication BF was the BF for the combined data of Experiments 2a and 2b and those of group few (BF_10_ = 2071) as a ratio of the BF of the combined data of Experiments 2a and 2b (BF_10_ = 69.74). Therefore, the replication BF_10_ = 29.70, suggesting that evidence for replication of the uncertainty effect was 29.70 times greater than for no effect of uncertainty. For the predictiveness effect, the replication BF was the BF for the combined data of Experiments 4a and 4b and those of group many (BF_10_ = 107027) as a ratio of the BF for the combined data of Experiments 4a and 4b (BF_10_ = 216.7). Therefore, the replication BF_10_ = 493.89, suggesting that evidence for replication of the predictiveness effect was 493.89 times greater than for no effect of predictiveness.

The results of Experiment 5 replicate the findings of Experiments 2a and 2b and Experiments 4a and 4b. When participants were trained with four uncertain compounds uncertain cues were learned more readily than predictive cues, but the pattern was the opposite when the number of uncertain cues was increased to eight cues. This suggests that the crucial difference between the results of Experiments 2a and 2b and 4a and 4b and those of Livesey et al. (2011) was the difficulty of the Stage 1 training determined by the number of trials that were impossible to learn. Importantly, the results show that the uncertainty effect that was observed when there were few uncertain compounds is robust. This effect was observed in Experiments 2a, 2b, and 5 (group few). Similarly, the predictiveness effect that was observed when there were eight uncertain compounds is also robust and was observed repeatedly in Experiments 4a, 4b, and 5 (group many).

## Experiment 6

The collective results of Experiments 2 through 5 suggest that attention is greater for uncertain cues than predictive cues when participants are required to learn about four uncertain cues, but this pattern switches when the number is eight uncertain cues. A number of factors are affected by the increase in uncertain cues. Thus, because of the increase in trial types, the average intertrial interval between repetitions of the same trial-type is longer. The overall memory load in terms of the total number of cues is also increased. Although the predictiveness effect was observed in Experiments 4 and 5 when the number of uncertain cues was increased, it is possible that an increase in cues generally may be sufficient to cause the predictiveness effect. This was tested in Experiment 6. Participants received a procedure like that used in Experiment 4b and for group many in Experiment 5. However, instead of receiving training with eight uncertain compounds, they received training with four uncertain compounds and four extra compounds consisting of one predictive cue and one irrelevant cue. If associability is higher for predictive cues than uncertain cues in Experiments 4 and 5 because of the increase in memory load (i.e., number of cues) and/or increased temporal spacing of trial types then four extra predictive/irrelevant compounds should also lead to increase in associability of predictive cues relative to uncertain cues.

### Method

#### Participants

Twenty-four people (17 women, seven men) participated in Experiment 6. The age range was 19 to 26 (*M* = 21.08, *SD* = 2.08). All other details were the same as Experiment 1.

#### Apparatus and stimuli

The apparatus and stimuli were the same as Experiment 4b.

#### Procedure

The procedure was the same as Experiment 4b and the many condition in Experiment 5 except that the compounds ZM, ZO, NO, and NM during Stage 1 training were not uncertain cues, but now reliably led to particular outcomes (see Table 4). Specifically, ZM and ZO led to Outcome 1 and NO and NM led to Outcome 2.[Table-anchor tbl4]

### Results and Discussion

#### Stage 1

Participants acquired the discrimination over training with performance increasing for the predictive/irrelevant condition over blocks, but no improvement for the uncertain condition. Mean performance on the last block (four trials of each trial type) was 77.34% (*SEM* = 3.31) for the predictive/irrelevant condition and 45.31% (*SEM* = 2.79) for the uncertain condition. There was a significant effect of block, *F*(3, 69) = 4.85, *p* < .04, η_p_^2^ = .17, 90% CI [.04, .28], and a significant effect of condition, *F*(1, 23) = 83.31, *p* < .001, η_p_^2^ = .78, 90% CI [.61, .85]. There was a significant interaction between factors, *F*(3, 69) = 8.78, *p* < .001, η_p_^2^ = .28, 90% CI [.11, .38]. Four participants failed to show performance of 60% and above on the predictive/irrelevant condition in the last half of training, but all of them performed above 50% correct.

#### Stage 2

Participants acquired the discrimination over training for both the novel cues and the recombined cues from Stage 1. Mean performance on the last block (two trials of each trial type) was 80.73% (*SEM* = 3.45) for the recombined condition and 90.62% (*SEM* = 2.52) for the novel condition. There was a significant effect of block, *F*(3, 69) = 38.41, *p* < .001 η_p_^2^ = .63, 90% CI [.49, .69], but no significant main effect of condition, *F*(1, 23) = 1.18, *p* = .29, η_p_^2^ = .05, 90% CI [.00, .23]. There was no significant interaction of factors, *F*(3, 69) = 1.91, *p* = .14, η_p_^2^ = .08, 90% CI [.00, .16].

#### Test

**Figure 7 fig7:**
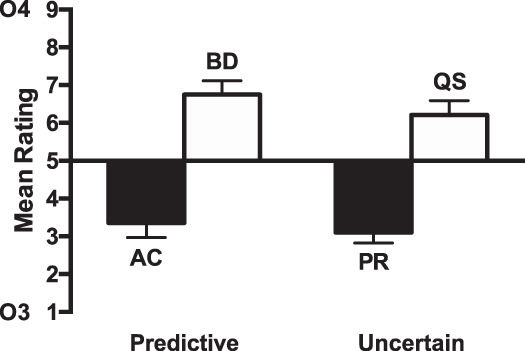
The results of the test phase of Experiment 6. The likelihood that Outcomes 3 and 4 would occur for each test compound was rated on a scale ranging from 1 to 9, with scores below 5 indicating an expectation that Outcome 3 would occur and scores above 5 indicating that Outcome 4 would occur. AC and BD were predictive cues paired with Outcomes 3 and 4, respectively. PR and QS were uncertain cues paired with Outcomes 3 and 4, respectively. Error bars indicate standard error of the mean.

The ratings for the test stage are shown in Figure 7. The ratings for compounds consisting of cues paired with Outcome 4 (BD, QS) were higher than for those paired with Outcome 3 (AC, PR), indicating that participants learned the cue–outcome associations. The difference between cues paired with Outcomes 3 and 4 was similar for the predictive and uncertain conditions. A 2 (predictiveness: predictive cues [AC, BD] vs. uncertain cues [PR, QS]) × 2 (Outcome: 3 [AC, PR] vs. 4 [BD, QS]) ANOVA was conducted. There was no significant effect of predictiveness, *F*(1, 23) = 1,23 = 2.63, *p* = .32, η_p_^2^ = .10, 90% CI [.00, .30], but there was a significant effect of outcome, *F*(1, 23) = 46.32, *p* < .001, η_p_^2^ = .67, 90% CI [.44, .77]. There was no significant interaction between factors, *F* < 1, *p* = .71. To assess whether the data provided evidence for the null hypothesis (i.e., no difference in the extent of learning between predictive and uncertain cues) a Bayesian *t* test (Cauchy scale = 0.707) was performed in JASP (Love et al., 2019) comparing the difference between the ratings for BD and AC and the difference for QS and PR. It was found that BF_10_ = 0.23, suggesting that the evidence favored the null hypothesis. In addition, given the failure to replicate the predictiveness effect using four extra predictive compounds rather uncertain compounds, we calculated a replication BF to assess the evidence for a lack of replication. We conducted Bayesian *t* tests comparing the difference between QS and PR with the difference between BD and AC. The replication BF was the BF for the combined data of Experiments 4a, 4b, and group many from Experiment 5 and Experiment 6 (BF_10_ = 10,224) as a ratio of the BF for the combined data of Experiments 4a, 4b, and group many from Experiment 5 (BF_10_ = 107027). Therefore, the replication BF was BF_10_ = 0.096, suggesting evidence for a lack of replication of the predictiveness effect in Experiment 6 was more than 10 times greater than the evidence for a replication of the predictiveness effect. A similar analysis was conducted to assess evidence for a failure to replicate the uncertainty effect observed in Experiment 2a, 2b, and group few in Experiment 5. The replication BF was the BF for the combined data of Experiments 2a, 2b, and group few from Experiment 5 and Experiment 6 (BF_10_ = 187) as a ratio of the BF for the combined data of Experiments 2a, 2b and group few from Experiment 5 (BF_10_ = 2071). Therefore, the replication BF was BF_10_ = 0.090, suggesting evidence for a lack of replication of the uncertainty effect in Experiment 6 was more than 11 times greater than the evidence for a replication of the uncertainty effect. Analysis of the test phase results excluding the participants that failed to meet the Stage 1 learning criterion showed a similar pattern of results (see Table S1 and Figure S5 in the online supplemental material).[Fig-anchor fig7]

The results failed to demonstrate that four extra predictive/irrelevant compounds resulted in greater associability of predictive cues over uncertain cues. Instead, learning for predictive and uncertain cues was similar in the test phase suggesting that both types of cues received similar levels of attention during training. This failure to find a difference between cues indicates that it is unlikely that the effect of increased number of uncertain cues in Experiment 4 and 5 (group many) was simply the result of increased memory load. Furthermore, it suggests that it was also not due to an increase in the average interval between repetitions of the same cue over trials (i.e., a trial spacing effect). The failure to find an effect may instead suggest that the crucial factor is the number of uncertain compounds, not just the number of cues per se. Although this is possible, we are cautious about this interpretation because the present experiment did not include a condition in which participants received four extra uncertain compounds, rather than predictive/irrelevant compounds, which would have acted as a positive control.

Although we did not find that four extra predictive/irrelevant compounds led to greater attention to predictive cues over uncertain cues as in Experiments 4 and 5, we also did not find the opposite effect, which we found in Experiments 2 and 5 (group few). Once again, we are cautious about making comparisons across experiments, but this suggests that the switch between greater attention to uncertain cues to greater attention to predictive cues depends on a number of factors such as the number of cues and whether it is possible to learn about those cues (i.e., impossible discriminations). These factors may both act to generally increase task difficulty, which may determine whether predictive or uncertain cues receive the most attention.

## Experiment 7

**Figure 8 fig8:**
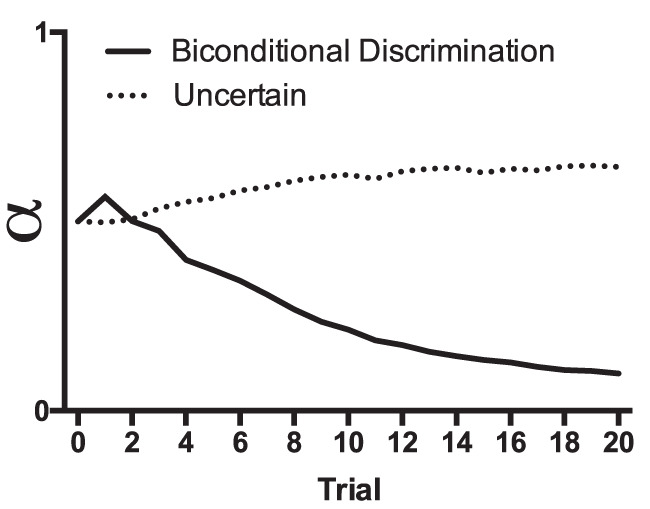
Simulation of changes in alpha across trials for cues used in a biconditional discrimination and uncertain cues. The biconditional discrimination used the design PQ+, RS+, PS−, and RQ−, in which “+” and “−” denote different outcomes. The same compounds were used for the uncertain cues, but each compound was equally paired with the two outcomes (i.e., PQ+/−, RS+/−, PS+/−, and RQ+/−). It was assumed that the unique configuration of the compound was represented by an additional cue (i.e., PQ^W^, RS^X^, PS^Y^, and RQ^Z^). The starting value of α was 0.5 for each cue. Lambda equaled 1, and S, the learning rate parameter determined by the outcome, was 0.3. The simulation was run using the CAL-R Pearce–Hall Simulator (Grikietis et al., 2016).

The results of Experiments 2a, 2b, and 5 demonstrate that uncertain cues increase in associability compared with predictive cues in particular circumstances. The results are consistent with the predictions of the Pearce and Hall (1980) model that proposes that the effect of uncertainty on attention is determined by its use of a summed error term. That uncertain cues were learned more readily than irrelevant cues in Experiment 1 is also consistent with associability being determined by a summed error term, because though individual error was equated between uncertain and irrelevant cues in that experiment, the summed error in Stage 1 training was greater for uncertain than for irrelevant cues. To test further the role of summed error in determining uncertainty effects on attention we conducted Experiments 7 and 8. Following the design of the experiments by Livesey et al. (2011), in Experiment 7 participants were trained on a biconditional discrimination (Saavedra, 1975), in addition to being trained on four uncertain compounds. For the biconditional discrimination, the cues were presented in a similar manner as the uncertain cues in Experiments 1 through 6, but rather than each compound being equally paired with two outcomes (e.g., PQ-1/2, RS-1/2, PS-1/2, RQ-1/2), each compound reliably led to only one outcome (e.g., PQ-1, RS-1, PS-2, RQ-2). Therefore, similar to cues in the uncertain compounds used in Experiment 1–6, the individual cues in the biconditional discrimination were not predictive of the outcome because across compounds they were equally paired with each outcome. Consequently, the individual prediction error for each cue on any given trial was high. In contrast to uncertain compounds of cues, however, the summed error for the biconditional discrimination cues will decrease as learning increases over the course of training, because the biconditional discrimination is soluble, with each compound reliably leading to a particular outcome, whereas it is impossible to predict the outcome using the uncertain cues. Thus, the design of Experiment 7 seeks to manipulate the summed error of different stimulus compounds. Figure 8 shows the results of a simulation of the Pearce–Hall model for acquisition of a biconditional discrimination and changes in the associability (α) of biconditional discrimination and uncertain cues. It was assumed that each compound elicits a unique configural cue (see Wagner, 1971). This results in the solution to the biconditional discrimination due to the unique configural cues predicting the occurrence of one or the other outcome (e.g., PQ^W^, RS^X^, PS^Y^, and RQ^Z^). Across trials, alpha declines for the biconditional discrimination cues, but remains high for the uncertain cues. Therefore, similar to the difference between uncertain and irrelevant cues in Experiment 1, it would be expected that attention will be greater for uncertain cues than for cues used in a biconditional discrimination. This prediction was tested in Experiment 7.[Fig-anchor fig8]

### Method

#### Participants

Thirty-two people (26 women, 6 men; age range = 18–29) participated in Experiment 7 (*M* = 21.72, *SD* = 2.53) All other details were the same as Experiment 1.

#### Apparatus and stimuli

All details were the same as Experiment 1.

#### Procedure

In Stage 1 of Experiment 7, participants were trained on a biconditional discrimination consisting of four compounds (see Table 5). Compounds PQ and RS were paired with Outcome 1 and compounds PS and RQ were paired Outcome 2. In addition, they were trained with four uncertain compounds (ZM, ZO, NO, and NM) that equally led to Outcomes 1 and 2 across trials. In Stage 2, participants were trained with four compounds that each consisted of a biconditional discrimination cue and an uncertain cue. Compounds PZ and RN were paired with Outcome 3, and compounds QM and SO were paired with Outcome 4. In the test phase, participants were presented with the compounds that consisted of either biconditional discrimination cues (PR, QS) or irrelevant cues (ZN, MO). Compounds PR and ZN consisted of cues that had been paired with Outcome 3 in Stage 2 and compounds QS and MO consisted of cues paired with Outcome 4 in Stage 2. All other details were the same as Experiment 1.[Table-anchor tbl5]

### Results and Discussion

#### Stage 1

Participants acquired the discrimination over training with performance increasing for the biconditional discrimination over blocks, but no improvement for the uncertain condition. Mean performance on the last block (four trials of each trial type) was 62.70% (*SEM* = 3.48) for the biconditional discrimination and 48.83% (*SEM* = 1.72) for the uncertain condition. There was a significant effect of block, *F*(3, 93) = 6.13, *p* < .001, η_p_^2^ = .17, 90% CI [.05, .26], and a significant effect of condition, *F*(1, 31) = 17.30, *p* < .001 η_p_^2^ = .36, 90% CI [.14, .52]. There was a significant interaction between factors, *F*(3, 93) = 3.70, *p* < .014 η_p_^2^ = .11, 90% CI [.01, .19]. Seventeen participants failed to show performance of 60% and above on the biconditional discrimination condition in the last half of training. Eight of those participants failed to perform above 50% correct.

#### Stage 2

Participants acquired the discrimination over training for both the novel cues and the recombined cues from Stage 1. Mean performance on the last block (two trials of each trial type) was 78.52% (*SEM* = 2.71) for the recombined condition and 82.03% (*SEM* = 3.37) for the novel condition. There was a significant effect of block, *F*(3, 93) = 38.87, *p* < .001, η_p_^2^ = .56, 90% CI [.43, .63], and a significant main effect of condition, *F*(1, 31) = 8.72, *p* = .006, η_p_^2^ = .22, 90% CI [.04, .40]. The interaction between factors was not significant, *F*(3, 93) = 2.69, *p* = .061, η_p_^2^ = .08, 90% CI [.00, .16].

#### Test

**Figure 9 fig9:**
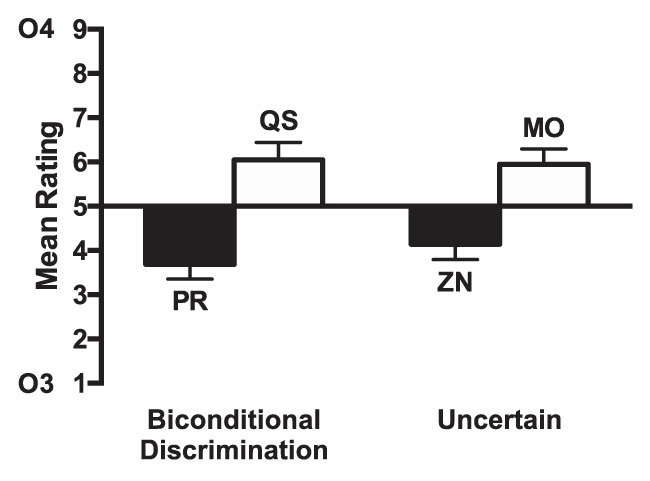
The results of the test phase of Experiment 7. The likelihood that Outcomes 3 and 4 would occur for each test compound was rated on a scale ranging from 1 to 9, with scores below 5 indicating an expectation that Outcome 3 would occur and scores above 5 indicating that Outcome 4 would occur. PR and QS were biconditional discrimination cues paired with Outcomes 3 and 4, respectively. ZN and MO were uncertain cues paired with Outcomes 3 and 4, respectively. Error bars indicate standard error of the mean.

The ratings for the test stage are shown in Figure 9. The ratings for compounds consisting of cues paired with Outcome 4 (MO, QS) were higher than for those paired with Outcome 3 (ZN, PR), indicating that participants learned the cue–outcome associations. The difference between cues paired with Outcome 4 and cues paired with Outcome 3 was similar for the biconditional discrimination cues and the uncertain cues. A 2 (predictiveness: biconditional discrimination [PR, QS] vs. uncertain [MO, ZN]) × 2 (Outcome: 3 [ZN, PR] vs. 4 [MO, QS]) ANOVA was conducted. There was a significant effect of outcome, *F*(1, 31) = 17.79, *p* < .001, η_p_^2^ = .36, 90% CI [.14, .53], but no significant effect predictiveness, *F* < 1, *p* = .48 or significant interaction of factors, *F* < 1, *p* = .44. In order to assess whether the data provided evidence for the null hypothesis (i.e., no difference in the extent of learning between predictive and uncertain cues) a Bayesian *t* test (Cauchy scale = 0.707) was performed comparing the difference between the ratings for QS and PR and the difference for ZM and MO. It was found that BF_10_ = 0.25, which suggests that evidence favored the null hypothesis. Analysis of the test phase results excluding the participants that failed to meet the Stage 1 learning criterion showed a similar pattern of results (see Table S1 and Figure S6 in the supplemental material).[Fig-anchor fig9]

The results of the test phase failed to demonstrate a significant difference in the associability of uncertain and biconditional discrimination cues. One explanation of the lack of difference between conditions is that it is possible that biconditional discrimination cues and uncertain cues received similar levels of attention in Stage 2 because of their training history in Stage 1. This possibility was explored in Experiments 8a and 8b. It is also possible, however, that the lack of difference between conditions was due to the large number of participants that failed to learn the biconditional discrimination to a sufficiently high level. Seventeen of the 32 participants did not meet the learning criterion of 60% correct and above in the last half of training. If participants failed to learn the biconditional discrimination, then the biconditional discrimination cues would be as equally nonpredictive of outcomes as uncertain cues. A reason for doubting whether a lack of learning led to the lack of difference in the associability of the cues is that when the analyses were restricted to the 15 participants that met the learning criterion Bayesian analysis still found evidence for the null hypothesis (BF_10_ < 0.33; see the online supplemental material). Therefore, a lack of difference in associability occurred despite successful learning of the biconditional discrimination.

## Experiments 8a and 8b

Experiment 7 failed to find a significant difference between the levels of associability of uncertain and biconditional discrimination cues. This may suggest that the associability of cues used in a biconditional discrimination undergo similar changes to uncertain cues. Therefore, it may not be the summed error of the compound of cues that determines changes in associability, but instead, the prediction error of individual cues independent of the associative strength of cues present on a trial. If attention to biconditional discrimination cues changes in a similar manner as for uncertain cues, then biconditional discrimination cues should function like uncertain cues in other circumstances. In order to test whether this is the case, Experiments 8a and 8b were replications of Experiments 2a and 4b (see also Experiments 2b and 5), but the four crucial uncertain cues from Stage 1 that went on to be tested were replaced with four cues used in a biconditional discrimination. In Experiment 8a participants were trained on the biconditional discrimination as well as the predictive/irrelevant compounds in Stage 1. If biconditional discrimination cues are similar to uncertain cues then it would be expected that, under these circumstances, biconditional discrimination cues will be paid more attention than predictive cues in Stage 2. Experiment 8b was similar to Experiment 8a, but there were four additional uncertain compounds in Stage 1. These additional uncertain compounds led to attention being paid to predictive cues rather than uncertain cues in Experiments 4 and 5. If biconditional discrimination cues are similar to uncertain cues then it would be expected that, in this situation, biconditional discrimination cues will be paid less attention than predictive cues.

### Method

#### Participants

Twenty-one people (15 women, 6 men; age range = 20–35) participated in Experiment 8a (*M* = 24.76, *SD* = 3.28) and 24 (20 women, 4 men; age range = 18–36) in Experiment 8b (*SEM* = 20.91, *SD* = 4.04). All other details were the same as Experiment 1.

#### Apparatus and stimuli

All details were the same as Experiment 1.

#### Procedure

In Stage 1 of Experiment 8a, participants were trained on a biconditional discrimination in a similar manner to Experiment 7 (see Table 6). They also received training with eight compounds that consisted of one cue that was predictive of the outcome (A–D) over trials and one cue that was not predictive over trials (i.e., irrelevant cues: W–Y). In Stage 2, participants received training with compounds that consisted of one biconditional discrimination cue and one predictive cue. Compounds AP and CR were paired with Outcome 3 and compounds BQ and DS were paired with Outcome 4. In the test phase, participants were presented with compounds that consisted of either biconditional discrimination cues (PR, QS) or predictive cues (AC, BD). Compounds PR and AC consisted of cues previously paired with Outcome 3 in Stage 2 and compounds QS and BD consisted of cues previously paired with Outcome 4 in Stage 2. All other details were the same as Experiment 1. Experiment 8b was the same as Experiment 8a except that in Stage 1 participants received training with four extra uncertain compounds (ZM, ZO, NM, NO) that were equally often paired with Outcomes and 1 and 2 (see Table 6).[Table-anchor tbl6]

### Results

#### Experiment 8a

##### Stage 1

Participants acquired the discrimination with performance increasing for the biconditional discrimination and the predictive/irrelevant condition over blocks. Mean performance on the last block (four trials of each trial type) was 79.61% (3.76 *SEM*) for the predictive/irrelevant condition and 79.17% (*SEM* = 3.49) for the biconditional discrimination condition. There was a significant effect of block, *F*(3, 60) = 28.80, *p* < .001, η_p_^2^ = .59, 90% CI [.43, .67], but no significant effect of condition, *F*(1, 20) = 2.57, *p* = .12, η_p_^2^ = .11, 90% CI [.00, .33], or interaction of factors, *F* < 1, *p* = .43. Five participants failed to show performance of 60% and above on the biconditional discrimination condition in the last half of training. Two of those participants failed to perform above 50% correct.

##### Stage 2

Participants acquired the discrimination over training for both the novel cues and the recombined cues from Stage 1. Mean performance on the last block (two trials of each trial type) was 86.31% (*SEM* = 3.75) for the recombined condition and 84.52% (*SEM* = 3.65) for the novel condition. There was a significant effect of block, *F*(3, 60) = 26.69, *p* < .001, η_p_^2^ = .57, 90% CI [.41, .65], but no significant main effect of condition, *F* < 1, *p* > .99. The interaction between factors was not significant, *F* < 1, *p* = .58.

##### Test

**Figure 10 fig10:**
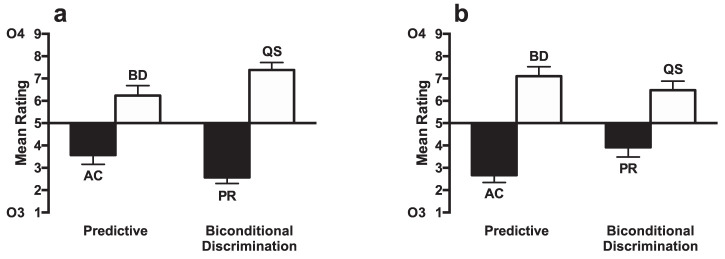
The results of the test phase of Experiment 8a (Panel a) and 8b (Panel b). The likelihood that Outcomes 3 and 4 would occur for each test compound was rated on a scale ranging from 1 to 9, with scores below 5 indicating an expectation that Outcome 3 would occur and scores above 5 indicating that Outcome 4 would occur. AC and BD were predictive cues paired with Outcomes 3 and 4, respectively. PR and QS were biconditional discrimination cues paired with Outcomes 3 and 4, respectively. Error bars indicate standard error of the mean.

The ratings for the test stage are shown in Figure 10a. The ratings for compounds consisting of cues paired with Outcome 4 (BD, QS) were higher than for those paired with Outcome 3 (AC, PR), indicating that participants learned the cue–outcome associations. The difference between cues paired with outcome and 4 and cues paired with Outcome 3 was greater for the biconditional discrimination cues than the predictive cues. A 2 (predictiveness: biconditional discrimination [PR, QS] vs. predictive [AC, BD]) × 2 (Outcome: 3 [AC, PR] vs. 4 [BD, QS]) ANOVA was conducted. There was a significant effect of outcome, *F*(1, 20) = 59.39, *p* < .001, η_p_^2^ = .75, 90% CI [.54, .83], but no significant effect predictiveness, *F* < 1, *p* = .78. There was a significant predictiveness by outcome interaction, *F*(1, 20) = 8.74, *p* = .008, η_p_^2^ = .30, 90% CI [.05, .50], indicating that the effect of outcome was significantly greater for the biconditional discrimination condition than for the predictive condition. Simple main effects analysis revealed that the effect of outcome was significant for both biconditional discrimination cues, *F*(1, 20) = 82.17, *p* < .001, η_p_^2^ = .80, 90% CI [.63, .86], and predictive cues, *F*(1, 20) = 15.74, *p* = .001, η_p_^2^ = .44, 90% CI [.15, .61]. Analysis of the test phase results excluding the participants that failed to meet the Stage 1 learning criterion showed a similar pattern of results (see Table S1 and Figure S7 in the online supplemental material).[Fig-anchor fig10]

#### Experiment 8b

##### Stage 1

Participants acquired the discrimination with performance increasing for the biconditional discrimination and the predictive/irrelevant condition over blocks but no improvement for the uncertain condition. Mean performance on the last block (four trials of each trial type) was 71.87% (*SEM* = 3.36) for the predictive/irrelevant condition and 68.53% (*SEM* = 3.43) for the biconditional discrimination condition, and 40.36% (*SEM* = 2.55) for the uncertain condition. There was a significant effect of block, *F*(3, 69) = 7.84, *p* < .001 η_p_^2^ = .25, 90% CI [.10, .36], and a significant effect of condition, *F*(2, 46) = 49.45, *p* < .001, η_p_^2^ = .68, 90% CI [.53, .75], and a significant interaction of factors, *F*(6, 138) = 6.84, *p* < .001, η_p_^2^ = .23, 90% CI [.11, .30]. A separate ANOVA restricted just to the predictive/irrelevant and biconditional discrimination conditions failed to find an effect of condition, *F* < 1, *p* = .88 and a condition by block interaction, *F* < 1, *p* = .45. Nine participants failed to show performance of 60% and above on the biconditional discrimination condition in the last half of training, but all of them performed above 50% correct.

##### Stage 2

Participants acquired the discrimination over training for both the novel cues and the recombined cues from Stage 1. Mean performance on the last block (two trials of each trial type) was 84.90% (*SEM* = 3.19) for the recombined condition and 89.68% (*SEM* = 3.15) for the novel condition. There was a significant effect of block, *F*(3, 69) = 30.48, *p* < .001, η_p_^2^ = .60, 90% CI [.42, .65], but no significant main effect of condition, *F*(1, 23) = 2.47, *p* = .13, η_p_^2^ = .10, 90% CI [.00, .30]. The interaction between factors was not significant, *F* < 1, *p* = .63.

##### Test

The ratings for the test stage are shown in Figure 10b. The ratings for compounds consisting of cues paired with Outcome 4 (BD, QS) were higher than for those paired with Outcome 3 (AC, PR), indicating that participants learned the cue–outcome associations. The difference between cues paired with Outcome 4 and cues paired with Outcome 3 was greater for the predictive cues than the biconditional discrimination cues. A 2 (predictiveness: biconditional discrimination [PR, QS] vs. predictive [AC, BD]) × 2 (Outcome: 3 [AC, PR] vs. 4 [BD, QS]) ANOVA was conducted. There was a significant effect of outcome, *F*(1, 23) = 60.10, *p* < .001, η_p_^2^ = .72, 90% CI [.52, .81], but no significant effect predictiveness, *F*(1, 23) = 1.24, *p* = .28, η_p_^2^ = .05, 90% CI [.00, .23]. There was a significant predictiveness by outcome interaction, *F*(1, 23) = 4.70, *p* = .041, η_p_^2^ = .17, 90% CI [.004, .37]. Simple main effects analysis revealed that the effect of outcome was significant for both biconditional discrimination cues, *F*(1, 23) = 15.92, *p* < .001, η_p_^2^ = .41, 90% CI [.14, .58], and predictive cues, *F*(1, 23) = 53.39, *p* < .001, η_p_^2^ = .70, 90% CI [.48, .79]. Analysis of the test phase results excluding the participants that failed to meet the Stage 1 learning criterion showed a similar pattern of results (see Table S1 and Figure S7 in the online supplementary material).

### Discussion

Experiments 8a and 8b demonstrated that biconditional discrimination cues functioned in a similar way to uncertain cues. The associability of biconditional discrimination cues depended on the total number of compounds that consisted of cues that individually were not predictive of the outcome. Thus, in Experiment 8a in which participants were trained with four biconditional discrimination cues and no extra uncertain cues, participants learned more about biconditional discrimination cues than predictive cues. In contrast, in Experiment 8b, in which participants were also trained with four extra uncertain cues, participants learned more about predictive cues than biconditional discrimination cues.

Across Experiments 8a and 8b biconditional discrimination cues functioned in a similar manner to uncertain cues in that they were learned more readily than predictive cues in Stage 2 when there were four uncertain compounds and the opposite was true when there were eight uncertain compounds. This may suggest that the ability of the configuration of cues to predict the outcome does not affect the attention paid to cues that individually are not predictive. Therefore, these results are contrary to our predictions based on the results of Experiment 1 and the predictions of the Pearce and Hall (1980) model, that the summed error of a compound determines the effect of uncertainty on attention. Instead, the results suggest that the individual prediction error of cues, independent of the summed error of the compound, determines uncertainty and predictiveness effects. Although it is possible that the summed error of biconditional discrimination compounds and predictive/irrelevant compounds may have differed, depending on the assumptions of how configural discriminations are learned (e.g., Pearce, 1987; Rescorla, 1973; Wagner, 2003), there was no significant difference in the accuracy of performance across blocks for the two conditions in both experiments. Therefore, this suggests that both discriminations were learned to a similar extent over training and that differences in the test are unlikely due to differences in the summed error of the compounds.

The similar levels of acquisition of the predictive/irrelevant discrimination and the biconditional discrimination are, however, surprising because it is typically found that biconditional discriminations are harder to learn than simple, nonconfigural discriminations in which individual cues reliably lead to an outcome (Livesey, Don, Uengoer, & Thorwart, 2019; Livesey et al., 2011; Saavedra, 1975). Indeed, biconditional discriminations are usually found to be harder to learn than other nonlinear configural discriminations such as the negative patterning discrimination (Harris & Livesey, 2008). It is not clear what the cause of the failure to observe a difference between conditions may be, but in comparison to Livesey et al. (2011) who did find a difference using procedures similar to those in Experiment 8, it is noticeable that in their study acquisition of the predictive/irrelevant condition was superior to acquisition in Experiments 8a and 8b. Therefore, the lack of difference between the predictive/irrelevant condition and biconditional discrimination condition in the current experiment may be due to the surprisingly low level of learning in the predictive/irrelevant condition rather than a surprisingly high level of learning on the biconditional discrimination. The relatively poor performance on the predictive/irrelevant condition may mask a difference between the conditions. It is also possible that the lack of difference may be due to participants encoding the compounds in a configural manner in both conditions even though the predictive/irrelevant condition did not require a configural solution (Alvarado & Rudy, 1992; Astur & Sutherland, 1998; Healey & Gaffan, 2001; Shanks & Darby, 1998). This seems unlikely, however, because this would have led to no advantage of one type of cue (predictive and biconditional) over the other in the test phase which clearly was not the case.

## General Discussion

Across Experiments 2a, 2b, and 5 (group few) participants learned more about cues that had previously led to an uncertain outcome compared with cues that were predictive of an outcome. The results provide a robust demonstration of uncertainty having a positive effect on associability in a learning task in humans. In contrast, Experiments 4a, 4b, and 5 (group many) demonstrated the opposite pattern of results, and participants learned more about predictive cues than uncertain cues. These results are more in line with previous work demonstrating a role for predictiveness in human associative learning (Le Pelley et al., 2016). Importantly, the key factor that determined whether an uncertainty effect (Experiments 2 and 5) or a predictiveness effect (Experiment 4 and 5) was observed was the number of uncertain cues that participants were required to learn about. When the number was four, an uncertainty effect was found, but when it increased to eight, a predictiveness effect was found. The effect of the number of uncertain cues was directly tested in Experiment 5 and it was found that the number determined the nature of the change in associability that occurred because of learning. Furthermore, the number of compounds consisting of two cues that were individually nonpredictive of outcomes also determined whether biconditional discrimination cues or cues that were individually predictive of outcomes increased in associability. Thus, in Experiment 8a, when the number of compounds was four, participants learned more about biconditional discrimination cues than predictive cues and in Experiment 8b, the opposite was true when the number of compounds was eight.

The finding that uncertain cues increased in associability relative to predictive cues in Experiments 2a, 2b and 5 was in contrast to results of Livesey et al. (2011). The cause of the difference between our results and theirs was not the nature of stimuli or cover story (Experiment 2b) and not the manner in which cues were combined in Stage 2 and in the test phase (Experiment 4a and 4b). Instead, it was the number of uncertain cues that were presented in Stage 1 training. As mentioned in the preceding text, the effect of the number of uncertain cues also determined whether participants learned more about biconditional cues or predictive cues. Therefore, the number of uncertain cues was repeatedly found to be a key determinant of whether an uncertainty or predictiveness effect was found.

The current results demonstrate that uncertainty can affect the associability of cues indicating that uncertain cues received more processing than predictive cues in certain conditions. Changes in associability reflect changes in attentional resources. In the current experiments it is not known whether uncertainty affected other measures of attention. A study by Beesley et al. (2015) provided some evidence that overt attention, as measured by eye gaze, is greater for uncertain cues than predictive cues. In that study, however, increased overt attention did not translate into greater associability of cues. Therefore, overt attention did not necessarily result in greater processing and encoding of information. Although associability is an indirect measure of attention generally, its advantage over other measures it that it provides a measure of the depth of selective processing. It remains to be seen whether uncertainty as manipulated in the current study affects other measures of selective attention.

### The Role of Task Difficulty in Modulating Associability

The role of the number of uncertain cues suggests that it is the level of task difficulty that determines whether uncertain cues or predictive cues are paid more attention. Experiment 6 was designed to test the nature of the task difficulty. In that experiment participants received additional compounds that should increase the overall memory load, but in contrast to Experiments 4 and 5 the extra compounds included a cue that was predictive and one cue that was irrelevant rather than consisting of two nonpredictive cues. In the test phase, participants showed similar levels of learning for uncertain and predictive cues, suggesting that the increase in the number of cues was not sufficient to switch attention toward predictive cues. Equally, the failure to find an uncertainty effect may suggest that the additional compounds made the task sufficiently difficult to not see such an effect. Although we are cautious about drawing conclusions from a null result, the results suggest that the crucial factor for determining whether attention switches to predictive cues is the increase in the number of uncertain cues. Therefore, both the cognitive load and the number of uncertain cues must be high.

If the number of uncertain compounds affects cognitive load then it may be expected that learning in the first stage of the procedure should be poorer when participants receive the more complex procedure with eight uncertain compounds compared with those that receive only found uncertain compounds. We failed to observe this effect in Experiment 5 in which performance under both procedures (four or eight uncertain compounds) was directly compared. In order to provide a more robust test of this prediction we pooled the data across the experiments that used four uncertain compounds (Experiments 2a, 2b, and condition few of Experiment 5) and those that used eight uncertain compounds (Experiments 4a, 4b, and condition many of Experiment 5) and compared performance on the predictive/irrelevant condition across the four blocks of Stage 1. Mean performance across Blocks 1 through 4 was 59%, 68%, 72%, and 77% correct for the four uncertain compounds condition and 58%, 62%, 68%, and 69% correct for the eight uncertain compounds condition. It was found that there was significant Condition (four vs. eight uncertain compounds) × Block interaction, *F*(3, 480) = 4.41, *p* = .007, η_p_^2^ = .03, 90% CI [.005, .05]. These results suggest that the number of uncertain cues did affect the ease at which learning occurred for the predictive/irrelevant condition. It is important to note that in Experiment 6, in which neither an uncertainty nor predictiveness effect was observed, performance in the last block of Stage 1 training for was 77% correct. This level of performance is like that found across the combined data of Experiments 2a, 2b, and 5 (few condition) in which an uncertainty effect was found (see the preceding text). Although we are cautious about drawing conclusions across experiments, this may suggest that an increase in cues in Experiment 6 was sufficient to abolish an uncertainty effect in the test phase but not sufficient to reduce levels of accuracy in Stage 1 training. Although we have described memory load as a factor that affects task difficulty, increasing memory load does not necessarily reduce accuracy on the Stage 1 discrimination. This could be for several reasons. For example, there may be different thresholds for an effect of memory load on accuracy and on associability. Nonetheless, the pattern of data across experiments suggests that manipulations of task complexity (memory load and the uncertainty of the cues) has a greater effect on associability than on accuracy of learning.

The switch between the uncertainty effect and the predictiveness effect may reflect the effect of task difficulty on controlled attention. Controlled attention will be engaged in order to reduce uncertainty (Pearce & Hall, 1980; Schneider & Shiffrin, 1977; Shiffrin & Schneider, 1977), but the ability to do this may be weakened as task difficulty increases. In contrast, attention paid to predictive cues may reflect a more automatic form of attention (Le Pelley, Vadillo, & Luque, 2013; Luque, Vadillo, Le Pelley, & Beesley, 2017; but see Mitchell, Griffiths, Seetoo, & Lovibond, 2012, for a potential role of controlled processing in the predictiveness effect). When task difficulty is low the effect of controlled attention paid to uncertain cues may be greater than the automatic attention paid to predictive cues, but as task difficulty increases the cognitive resources required to engage controlled attention are decreased such that automatic attention wins out over controlled attention. In other words, when there is a low level of difficulty participants actively engage in a strategy of exploration of cues in order to resolve uncertainty, but when there is a higher level of difficulty participants switch to a less effortful strategy of exploitation of predictive cues.

Another possibility is that the task difficulty influences the extent to which uncertainty is surprising or instead, expected (Behrens, Woolrich, Walton, & Rushworth, 2007; Yu & Dayan, 2005). For example, Easdale et al. (2017) manipulated the overall degree of uncertainty in a learning task and then, in a second stage, examined the ability of participants to learn new associations that led to particular outcomes in a probabilistic manner. For some participants, the level of uncertainty in the second stage was surprising (i.e., it was greater than previously experienced), whereas for other participants the level of uncertainty across both stages of training was the same. It was found that participants that experienced a surprising level of uncertainty learned faster in the second stage than participants that received a constant level of uncertainty. In the present experiments, it is possible that a large number of uncertain cues increases the experience of uncertainty such that it is expected. This may lead to a reduction in the attention paid to uncertain cues. When the number of uncertain cues is low or they are relatively scarce compared with predictive cues, then uncertainty is still surprising and, therefore, attention remains high. The results of Experiment 6, in which it was found that there was no significant advantage of uncertain cues over predictive cues when the number of predictive cues was increased, suggests that the number or proportion of uncertain cues cannot be the only factor that determines whether uncertainty is surprising. Thus, in Experiment 6, the uncertain cues were relatively scarce compared with predictive cues. This should have led to uncertain cues being particularly surprising, but an uncertainty effect was not observed.

### Changes in Associability Depend on the Individual Prediction Error of Cues

Cues that were uncertain by virtue of being involved in a biconditional discrimination underwent changes in associability in a similar manner to uncertain cues that were presented in compounds that were not predictive of outcomes (Experiments 7 and 8). This finding is in contrast to work demonstrating that cues used in nonlinear configural discriminations are paid attention on the basis of whether they are relevant for learning (George & Pearce, 1999; Kruschke, 1996). In the present experiments, the biconditional discrimination cues were relevant, but whether they received greater attention than predictive cues or not depended on the level of task difficulty. The fact that the associability of biconditional discrimination cues changed in a manner that was similar to uncertain cues suggests that the effect of uncertainty on attention that was observed in Experiments 2 and 5 was not due to the summed error of the compounds. By the end of Stage 1 training, summed error should be low for biconditional discrimination cues and high for uncertain cues. This contradicts the proposal of the Pearce and Hall (1980) model that assumes that the summed error, reflecting the discrepancy between the outcome and the combined predictive strength of all the cues that are present on a trial, is positively related to the amount of attention that cues receive. The lack of difference between uncertain and biconditional cues, instead, suggests that it is the individual prediction error for each cue, independent of the other cues that are present, that determines the level of attention of attention that the cue receives in both circumstances in which individual prediction error leads to a reduction in associability relative to predictive cues (see also Livesey et al., 2019; Livesey et al., 2011) and increase in associability. It is important to note that the summed error of compounds in a biconditional discrimination cannot simply reflect the summed error of the two individual cues. The two individual cues are uncertain due to being paired with Outcomes 1 and 2 equally, and therefore, their combined associative strength will be equally nonpredictive of the particular outcomes. Therefore, the summed error that reduces as biconditional discrimination learning increases must reflect the associative strength of the aspects of the compound that reflect the unique configuration of the two cues (Pearce, 1987; Rescorla, 1973; Wagner, 2003). This means that though the individual error of cues used in a biconditional discrimination is not involved in the mechanism required for biconditional discrimination learning, our results suggest that it is involved in the mechanisms for changes in the associability of cues.

Although the results of Experiments 8a and 8b suggest that it is the individual prediction error of cues that drives changes in attention, the results of Experiment 1 do not fit with this claim. In Experiment 1 uncertain cues were learned more readily than irrelevant cues. Both uncertain and irrelevant cues were nonpredictive of outcomes, but irrelevant cues were presented in compound with predictive cues whereas uncertain cues were presented in compound with other uncertain cues. Therefore, the cues were matched for individual error, but the summed error of compounds was lower for irrelevant cues than for uncertain cues. The advantage of uncertain cues over irrelevant cues suggest that it is the summed error of the compound that affects attention, a conclusion that is line with the Pearce and Hall model (1980). The reasons for the discrepancy between the results of Experiment 1 and those of Experiments 8a and 8b are not clear, but one possibility is that irrelevant cues are subject to performance effects that do not apply to uncertain cues. It is possible that in Stage 1 participants learned to ignore irrelevant cues due to the presence of the predictive cues and this avoidance response carried over to the Stage 2 training. This would have resulted in participants preferentially looking at uncertain cues over irrelevant cues. It has been demonstrated that participants orient toward predictive cues even when there is no instrumental contingency between looking at the cue and the occurrence of the outcome (Le Pelley, Pearson, Griffiths, & Beesley, 2015), suggesting that the orienting response is controlled by Pavlovian associations. It is possible that a reciprocal avoidance response is also acquired to irrelevant stimuli. There are a number of demonstrations that stimuli that are irrelevant (either by virtue of being nonpredictive and presented in compound with predictive stimuli or by being a redundant predictor) receive less overt attention than predictive cues (Beesley & Le Pelley, 2011; Le Pelley, Beesley, & Griffiths, 2011; Luque, Vadillo, Gutierrez-Cobo, & Le Pelley, 2018), but it is not clear whether this is due to an increase in attention to predictive cues or due to a reduction in attention to irrelevant cues. Nonetheless, it is clear that irrelevant cues, as a consequence of being presented in compound with predictive cues, may be subject to a number of processes that contribute to a reduction in attention, in a manner that is different to uncertain cues. Although this appears to be case in the present study (Experiment 1), a difference in the associability of irrelevant cues and uncertain cues is not always observed (Livesey et al., 2011). Whether task difficulty as determined by the number of uncertain compounds is the crucial factor that determines whether a difference between irrelevant cues and uncertain cues is observed remains to be seen.

### An Associability Versus an Interference Account of the Uncertainty Effect

An alternative account of the uncertainty effect that was observed in Experiments 2 and 5 with partially reinforced cues and in Experiment 8a with biconditional discrimination cues is that it reflects reduced interference from learning in Stage 1. If learning in Stage 1 interferes with learning in Stage 2 then it would be expected that there would be interference for predictive cues because they had formed a strong association with either Outcome 1 or 2 in Stage 1, but not for uncertain, partially reinforced or biconditional discrimination cues because they had not formed a strong association with a particular outcome. Any potential effect of interference, however, was reversed in Experiments 4, 5 (group many), and 8b in which it was found that individual cues that were predictive of outcomes in Stage 1 were learned about more readily in Stage 2. If the uncertainty effect was caused by interference then it must be assumed that the interference and predictiveness effects are in competition with one another and that an increase in the number of uncertain cues either reduces the interference effect or enhances the predictiveness effect. This may be unlikely because the strength of interference in Stage 2 should be related to how predictive a cue is in Stage 1. Thus, any manipulation of one factor should similarly affect the other factor. Therefore, it is not clear how an interference account of the uncertainty effect can be incorporated with the predictiveness effect that was also observed across experiments.

### Does the Predictiveness Effect Depend on Task Difficulty?

Our results suggest that if task difficulty is low then the chance of finding an uncertainty effect will be increased. There are, however, examples of predictiveness effects that may be hard to explain in terms of task difficulty (e.g., Kattner, 2015; Le Pelley, Turnbull, Reimers, & Knipe, 2010). In the study by Le Pelley, Turnbull, et al. (2010), a predictiveness effect was found even though, in comparison to our experiments, the design of the task was simple. In the first experiment participants were required to learn about six cues that were presented individually across trials. Four cues were predictive in Stage 1 training and two cues were uncertain by virtue of leading to two different outcomes equally often. In Stage 2 the cues were once again presented individually, and they now led to new outcomes with each cue now being predictive of the particular outcome with which it was paired. The test phase examined how much participants had learned about the cue–outcome associations in Stage 2. Therefore, any difference between the cues must reflect an effect of Stage 1 training on subsequent learning of new associations. It was found that participants learned more about the previously predictive cues than the uncertain cues. This procedure used fewer cues than our procedures and the number of uncertain cues was very low. Therefore, this finding does seem to contradict the idea that task difficulty influences attention. It is possible that when the design of tasks is very simple participants are more aware of the contingencies between cues and outcomes and engage in processes, such as propositional reasoning, that may be qualitatively different to the processes that are engaged when the task is more complex. Tasks may have to have a sufficient level of complexity for performance to reflect lower level associative learning processes that are engaged in incremental trial and error learning. Although we can provide an explanation for why Livesey et al. (2011) observed a predictiveness effect yet similar, simpler, experimental procedures produced the opposite uncertainty effect in the present experiments, it remains to be seen to what extent task difficulty can account for the presence or absence of an uncertainty effect using procedures that differ substantially from the ones used in the present study.

## Conclusion

The present results suggest that attention is allocated to stimuli in a top-down manner based on the prediction error associated with a cue and the current level of task difficulty determined by the level of uncertainty. There are parallels between this idea and that of perceptual load on selective attention (Lavie, Hirst, de Fockert, & Viding, 2004), but here, the load reflects a specific type of information (uncertainty) rather than just the total number of cues. The task now for theories of learning and attention is to understand the circumstances and decision-making computations that determine the switch between attention for uncertain and predictive cues. We have shown in a narrow range of situations that people do reliably flip between allocating attention to predictive and uncertain cues, but predicting which strategy is adopted in more complex situations requires greater understanding of the relevant task parameters. This, however, is important for determining when selective attention achieves efficient processing information and for when it leads to suboptimal selection of information. The balance between these processes is crucial for understanding the causes and consequences of impaired attention in psychopathology.

In conclusion, the present results suggest that uncertainty does play a role in determining attention paid to cues in human associative learning tasks. This provides the first clear link between the role of uncertainty in associative processes in animals and in humans. In contrast, however, to the theoretical analysis of uncertainty effects in animals (Pearce & Hall, 1980) our results suggest that uncertainty reflects the individual prediction error for cues rather than the summed prediction error. Furthermore, uncertainty is observed only under conditions in which the number of uncertain cues is low. Both uncertainty and predictiveness affect attention and the balance between the two processes is determined by the relative complexity/difficulty of the learning task.

## Supplementary Material

10.1037/xge0000991.supp

## Figures and Tables

**Table 1 tbl1:** Design of Experiments 1, 2a, and 2b and the Few Condition in Experiment 5

Stage 1	Stage 2	Test
	Experiment 1	
	VP-3	VX
Predictive/irrelevant	WQ-4	WY
AV-1	XR-3	PR
BV-2	YS-4	QS
AW-1	EF-3	EH
BW-2	GH-4	FG
CX-2	IJ-3	IJ
DX-1	KL-4	KL
CY-2		
DY-1	Experiments 2a, 2b, and 5 (few condition)	
Uncertain	AP-3	AC
PQ-1/2	BQ-4	BD
RQ-1/2	CR-3	PR
PS-1/2	DS-4	QS
RS-1/2	EF-3	EH
	GH-4	FG
	IJ-3	IJ
	KL-4	KL
*Note.* The table lists the trial types in each stage of the experiment. Letters denote cues; numbers denote outcomes (attack, retreat, support, surrender). Stage 1, in which participants received training with Predictive/irrelevant compounds and Uncertain compounds, was the same for Experiments 1, 2a, 2b and 5 (few condition). Stage 2 and test differed between Experiment 1 and Experiments 2a, 2b, and 5 (few condition) as indicated by the experiment headings.		

**Table 2 tbl2:** Design of Experiment 3

Stage 1	Stage 2	Test
AV-1	AX-3	AC
BV-2	BY-4	BD
AW-1	CV-3	VX
BW-2	DW-4	WY
CX-2	EF-3	EH
DX-1	GH-4	FG
CY-2	IJ-3	IJ
DY-1	KL-4	KL
*Note*. The table lists the trial types in each stage of the experiment. Letters denote cues; numbers denote outcomes (attack, retreat, support, surrender).		

**Table 3 tbl3:** Design of Experiments 4a and 4b and the Many Condition in Experiment 5

Stage 1	Stage 2	Test
	Experiment 4a	
Predictive/irrelevant	AP-3	AC
AV-1	BR-4	BD
BV-2	CZ-3	ZP
AW-1	DN-4	NR
BW-2	EF-3	EH
CX-2	GH-4	FG
DX-1	IJ-3	IJ
CY-2	KL-4	KL
DY-1		
Uncertain	Experiments 4b and 5 (many condition)	
PQ-1/2	AP-3	AC
RQ-1/2	BQ-4	BD
PS-1/2	CR-3	PR
RS-1/2	DS-4	QS
ZM-1/2	EF-3	EH
NM-1/2	GH-4	FG
ZO-1/2	IJ-3	IJ
NO-1/2	KL-4	KL
*Note.* The table lists the trial types in each stage of the experiment. Letters denote cues; numbers denote outcomes (attack, retreat, support, surrender). Stage 1, in which participants received training with Predictive/irrelevant compounds and Uncertain compounds, was the same for Experiments 4a, 4b, and 5 (many condition). Stage 2 and test differed between Experiment 4a and Experiments 4b and 5 (many condition) as indicated by the experiment headings.		

**Table 4 tbl4:** Design of Experiment 6

Stage 1	Stage 2	Test
Predictive/irrelevant	AP-3	AC
AV-1	BQ-4	BD
BV-2	CR-3	PR
AW-1	DS-4	QS
BW-2	EF-3	EH
CX-2	GH-4	FG
DX-1	IJ-3	IJ
CY-2	KL-4	KL
DY-1		
Uncertain		
PQ-1/2		
RQ-1/2		
PS-1/2		
RS-1/2		
Extra predictive/irrelevant		
ZM-1		
NM-2		
ZO-1		
NO-2		
*Note*. The table lists the trial types in each stage of the experiment. Letters denote cues; numbers denote outcomes (attack, retreat, support, surrender).		

**Table 5 tbl5:** Design of Experiment 7

Stage 1	Stage 2	Test
Biconditional discrimination	PZ-3	PQ
PQ-1	QM-4	RS
PS-2	RN-3	ZN
RS-1	SO-4	MO
RQ-2	EF-3	EH
Uncertain	GH-4	FG
ZM-1/2	IJ-3	IJ
ZO-1/2	KL-4	KL
NM-1/2		
NO-1/2		
*Note*. The table lists the trial types in each stage of the experiment. Letters denote cues; numbers denote outcomes (attack, retreat, support, surrender).		

**Table 6 tbl6:** Design of Experiment 8a and 8b

Stage 1	Stage 2	Test
Predictive/irrelevant	AP-3	AC
AV-1	BQ-4	BD
BV-2	CR-3	PR
AW-1	DS-4	QS
BW-2	EF-3	EH
CX-2	GH-4	FG
DX-1	IJ-3	IJ
CY-2	KL-4	KL
DY-1		
Biconditional discrimination		
PQ-1		
PS-2		
RS-1		
RQ-2		
Extra uncertain (Experiment 8b only)		
ZM-1/2		
NM-1/2		
ZO-1/2		
NO-1/2		
*Note*. The table lists the trial types in each stage of the experiment. Letters denote cues; numbers denote outcomes (attack, retreat, support, surrender). Stage 1 differed between Experiment 8a and 8b. Participants in both experiments received the predictive/irrelevant and biconditional discrimination trials. Participants in Experiment 8b only received the Extra uncertain trials.
