# Correction to Chao, McGregor, and Sanderson (2020)

**DOI:** 10.1037/xge0001032

**Published:** 2021-01-14

In the article “Uncertainty and Predictiveness Modulate Attention in Human Predictive Learning” by Chang-Mao Chao, Anthony McGregor, and David J. Sanderson (*Journal of Experimental Psychology: General*. Advance online publication. November 30, 2020. https://doi.org/10.1037/xge0000991), formatting for UK Research Councils funding was omitted. The author note and copyright line now reflect the standard acknowledgment of and formatting for the funding received for this article.

All versions of this article have been corrected.

