# Supplementary material for: Uncertainty and Predictiveness Modulate Attention in Human Predictive Learning
Source: J Exp Psychol Gen. 2020 Nov 30;150(6):1177–202. doi: 10.1037/xge0000991 (PMC8515774; doi:10.1037/xge0000991)
Supplement: Supplementary file 1 [file xge0000991.docx]

**Supplementary Material**

**Uncertainty and predictiveness modulate attention in human predictive learning**

Chang-Mao Chao, Anthony McGregor & David J. Sanderson

Table S1. Statistical analyses of the test phase of Experiments 1-8 that exclude the data from participants that were less than 60% accurate during the last half of training in stage 1 (see main text for details). The data of participants that met the learning criterion are shown in Figure S1 (Experiments 1, 2a and 2b), Figure S2 (Experiment 3), Figure S3 (Experiments 4a and 4b, Figure S4 (Experiment 5), Figure S5 (Experiment 6), Figure S6 (Experiment 7) and Figure S7 (Experiments 8a and 8b). Bayes factors are provided for Experiments 6 and 7, the two experiments that provided evidence for the null hypothesis when all participants were included (see main text). The results of these analyses are similar to those reported in the main text.

| *Experiment* | *Interaction* | *F* | *df* | *p* | *η_p_^2^ [90% CI]* | *BF_10_* |
| --- | --- | --- | --- | --- | --- | --- |
| 1 | Cue condition by outcome | 15.34 | 1,21 | 0.001 | 0.42 [0.14, 0.59] | - |
| 2a | Cue condition by outcome | 12.13 | 1,24 | 0.002 | 0.34 [0.09, 0.52] | - |
| 2b | Cue condition by outcome | 3.29 | 1,23 | 0.083 | 0.13 [0.00, 0.33] | - |
| 3 | Cue condition by outcome | 7.43 | 1,14 | 0.016 | 0.35 [0.04, 0.56] | - |
| 4a | Cue condition by outcome | 10.75 | 1,16 | 0.005 | 0.40 [0.09, 0.59] | - |
| 4b | Cue condition by outcome | 5.65 | 1,16 | 0.030 | 0.26 [0.01, 0.49] | - |
| 5 | Cue condition by outcome by group | 20.68 | 1,34 | < 0.001 | 0.38 [0.16, 0.53] | - |
| 6 | Cue condition by outcome | < 1 | 1,19 | 0.43 | - | 0.31 |
| 7 | Cue condition by outcome | < 1 | 1,14 | 0.82 | - | 0.27 |
| 8a | Cue condition by outcome | 4.57 | 1,15 | 0.049 | 0.23 [0.003, 0.47] | - |
| 8b | Cue condition by outcome | 7.04 | 1,15 | 0.018 | 0.32 [0.03, 0.54] | - |

**Figure S1**. The results of the test phase of Experiments 1, 2a and 2b in panels a-c respectively, excluding the data from participants that were less than 60% accurate during the last half of stage 1 in the predictive/irrelevant condition. The likelihood that outcomes 3 and 4 would occur for each test compound was rated on a scale from 1-9 with scores below 5 indicating an expectation that outcome 3 would occur and scores above 5 indicating that outcome 4 would occur. Panel a: VX and WY were irrelevant cues paired with outcomes 3 and 4 respectively. PR and QS were uncertain cues paired with outcomes 3 and 4 respectively. Panel b and c: AC and BD were predictive cues paired with outcomes 3 and 4 respectively. PR and QS were uncertain cues paired with outcomes 3 and 4 respectively. Error bars indicate SEM.

**Figure S2**. The results of the test phase of Experiment 3 excluding the data from participants that were less than 60% accurate during the last half of stage 1 in the predictive/irrelevant condition. The likelihood that outcomes 3 and 4 would occur for each test compound was rated on a scale from 1-9 with scores below 5 indicating an expectation that outcome 3 would occur and scores above 5 indicating that outcome 4 would occur. AC and BD were predictive cues paired with outcomes 3 and 4 respectively. VX and WY were irrelevant cues paired with outcomes 3 and 4 respectively. Error bars indicate SEM.

**Figure S3**. The results of the test phase of Experiments 4a and 4b in panels a and b respectively, excluding the data from participants that were less than 60% accurate during the last half of stage 1 in the predictive/irrelevant condition. The likelihood that outcomes 3 and 4 would occur for each test compound was rated on a scale from 1-9 with scores below 5 indicating an expectation that outcome 3 would occur and scores above 5 indicating that outcome 4 would occur. AC and BD were predictive cues paired with outcomes 3 and 4 respectively. In Experiment 5 (panel a), ZP and NR were uncertain cues paired with outcomes 3 and 4 respectively. In Experiment 6 (panel b), PR and QS were uncertain cues paired with outcomes 3 and 4 respectively. Error bars indicate SEM.

**Figure S4**. The results of the test phase of Experiment 5 excluding the data from participants that were less than 60% accurate during the last half of stage 1 in the predictive/irrelevant condition. The mean ratings for group few are on the left and those for group many are on the right. The likelihood that outcomes 3 and 4 would occur for each test compound was rated on a scale from 1-9 with scores below 5 indicating an expectation that outcome 3 would occur and scores above 5 indicating that outcome 4 would occur. AC and BD were predictive cues paired with outcomes 3 and 4 respectively. PR and QS were uncertain cues paired with outcomes 3 and 4 respectively. Error bars indicate SEM.

**Figure S5**. The results of the test phase of Experiment 6 excluding the data from participants that were less than 60% accurate during the last half of stage 1 in the predictive/irrelevant condition. The likelihood that outcomes 3 and 4 would occur for each test compound was rated on a scale from 1-9 with scores below 5 indicating an expectation that outcome 3 would occur and scores above 5 indicating that outcome 4 would occur. AC and BD were predictive cues paired with outcomes 3 and 4 respectively. PR and QS were uncertain cues paired with outcomes 3 and 4 respectively. Error bars indicate SEM.

**Figure S6**. The results of the test phase of Experiment 7 excluding the data from participants that were less than 60% accurate during the last half of stage 1 in the biconditional discrimination condition. The likelihood that outcomes 3 and 4 would occur for each test compound was rated on a scale from 1-9 with scores below 5 indicating an expectation that outcome 3 would occur and scores above 5 indicating that outcome 4 would occur. PR and QS were biconditional discrimination cues paired with outcomes 3 and 4 respectively. ZN and MO were uncertain cues paired with outcomes 3 and 4 respectively. Error bars indicate SEM.

**Figure S7**. The results of the test phase of Experiment 8a and 8b in panels a and b respectively, excluding the data from participants that were less than 60% accurate during the last half of stage 1 in the predictive/irrelevant condition. The likelihood that outcomes 3 and 4 would occur for each test compound was rated on a scale from 1-9 with scores below 5 indicating an expectation that outcome 3 would occur and scores above 5 indicating that outcome 4 would occur. AC and BD were predictive cues paired with outcomes 3 and 4 respectively. PR and QS were biconditional discrimination cues paired with outcomes 3 and 4 respectively. Error bars indicate SEM.
